# Cyanoacetohydrazide linked to 1,2,3-triazole derivatives: a new class of α-glucosidase inhibitors

**DOI:** 10.1038/s41598-022-11771-y

**Published:** 2022-05-23

**Authors:** Aida Iraji, Diba Shareghi-Brojeni, Somayeh Mojtabavi, Mohammad Ali Faramarzi, Tahmineh Akbarzadeh, Mina Saeedi

**Affiliations:** 1 Stem Cells Technology Research Center, Shiraz University of Medical Sciences, Shiraz, Iran; 2 Central Research Laboratory, Shiraz University of Medical Sciences, Shiraz, Iran; 3 Department of Medicinal Chemistry, Faculty of Pharmacy, Tehran University of Medical Sciences, Tehran, Iran; 4 Department of Pharmaceutical Biotechnology, Faculty of Pharmacy, Tehran University of Medical Sciences, P.O. Box 14155-6451, Tehran, 1417614411 Iran; 5 Persian Medicine and Pharmacy Research Center, Tehran University of Medical Sciences, Tehran, Iran; 6 Medicinal Plants Research Center, Faculty of Pharmacy, Tehran University of Medical Sciences, Tehran, Iran

**Keywords:** Structure-based drug design, Drug discovery and development

## Abstract

In this work, a novel series of cyanoacetohydrazide linked to 1,2,3-triazoles (**9a**–**n**) were designed and synthesized to be evaluated for their anti-α-glucosidase activity, focusing on the fact that α-glucosidase inhibitors have played a significant role in the management of type 2 diabetes mellitus. All synthesized compounds except **9a** exhibited excellent inhibitory potential, with IC_50_ values ranging from 1.00 ± 0.01 to 271.17 ± 0.30 μM when compared to the standard drug acarbose (IC_50_ = 754.1 ± 0.5 μM). The kinetic binding study indicated that the most active derivatives **9b** (IC_50_ = 1.50 ± 0.01 μM) and **9e** (IC_50_ = 1.00 ± 0.01 μM) behaved as the uncompetitive inhibitors of α-glucosidase with *K*_*i*_ = 0.43 and 0.24 μM, respectively. Moreover, fluorescence measurements were conducted to show conformational changes of the enzyme after binding of the most potent inhibitor (**9e**). Calculation of standard enthalpy (Δ*H*_m_°) and entropy (Δ*S*_m_°) values confirmed the construction of hydrophobic interactions between **9e** and the enzyme. Also, docking studies indicated desired interactions with important residues of the enzyme which rationalized the in vitro results.

## Introduction

Diabetes Mellitus (DM) is a common metabolic disease, characterizing by the hyperglycemia that impairs insulin production in the body. The global prevalence of DM and lacking definite treatment of the disease have become a challenging issue in the world^1^. Long-term dysfunction or failure of various body organs in patients with DM usually leads to severe complications such as kidney diseases, nervous system diseases, leg amputation, heart diseases, and blindness^1–3^. There are three main diabetes types, among which type 2 diabetes (T2DM) with over 85% of diabetics is known as the major type of DM^4,5^. The first-line medication in T2DM needs a reduction of hepatic glucose production through controlling the digestive enzyme activities or inhibition of carbohydrate digestive enzymes^6^.

α-Glucosidase (EC 3.2.1.20) is an exocyclic enzyme located in the epithelium of the human small intestine that hydrolyses the 1,4-α-glycosidic linkages of oligosaccharides and disaccharides to form monosaccharides. α-Glucosidase inhibitors slow down the digestion and absorption of simple carbohydrates in the intestine without direct effects on the secretion of insulin leading to the reduction of postprandial plasma glucose levels^7^. Noteworthy, α-glucosidase inhibitors are also ideal agents for other medical therapies such as hyperlipoproteinemia, obesity, and cancer^8–10^. Clinically approved α-glucosidase inhibitors to target T2DM named acarbose, miglitol, and voglibose have been used in the management of diabetes and obesity. Hence, α-glucosidase is an ideal target against T2DM and their inhibitors are used to alleviate the disease. However, the non-carbohydrate mimicking α-glucosidase inhibitors are limited. In the last years, a variety of synthetic and natural α-glucosidase inhibitors have been developed^11–16^. Most of the potent inhibitors contain heterocyclic compounds^11^ and coumarins^17^, thiadiazoles^18^, imidazoles and benzimidazoles^19,20^, pyrazoles-benzofurans^21^, oxindoles^22^, and isatins^16^ are examples of synthetic α-glucosidase inhibitors. Further, 1,2,3-triazole based compounds were recently introduced as potent α-glucosidase inhibitors^12,16,23,24^. 1,2,3-Triazole and its derivatives can be easily prepared through Click multicomponent reaction. The “click” in click chemistry refers to the rapid and selective reactions of small molecules leading to the formation of a wide range of products^25^. Among many click reactions described to date, copper (I)-catalyzed alkyne-azide cycloaddition introduced by Sharpless et al.^26^ has attracted much attention due to the potency of production of a library of bioactive 1,2,3-triazole derivatives through heteroatom links^27–32^. The α-glucosidase inhibitory activity of 1,2,3-triazoles have also been fully investigated in the literature^33^ as a prime choice for medicinal researchers to develop new anti-DM molecules.

In continuation of our efforts to develop novel and efficient anti-α-glucosidase compounds, cyanoacetohydrazide moiety was found to be an ideal and efficient pharmacophore by providing different interactions within the binding site of α-glucosidase. In this respect, new derivatives of cyanoacetohydrazide linked to 1,2,3-triazoles were designed and a library comprising of fourteen compounds was synthesized and evaluated for their in vitro α-glucosidase inhibitory activity. To investigate the interaction of these compounds with α-glucosidase, kinetic as well as molecular docking studies were also performed. Moreover, fluorescence measurements were recorded to characterize conformational changes of the enzyme after inhibition.

## Results and discussion

### Designing

**Figure 1:**
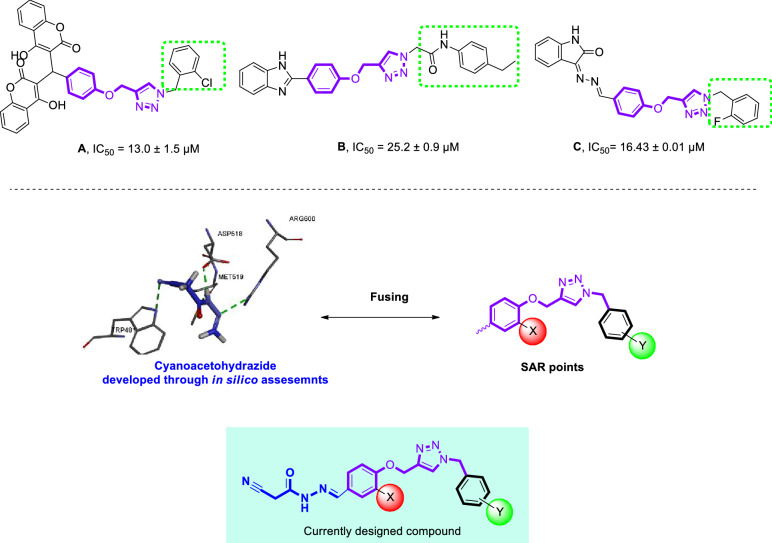
Design strategy for the SAR studies of current research.

Recently, a large number of research have focused on the physiological and therapeutic properties of benzyl-1,2,3-triazole moiety as a promising pharmacophore to design and develop new potentially useful therapeutic applications^23,28,34–37^. Recently, published data revealed that phenoxy-1,2,3-triazole-based scaffolds possessing benzyl substituents are important anti-α-glucosidase agents. For instance, Mahdavi et al. evaluated biscoumarin-phenoxytmethyltriazole derivatives as α-glucosidase inhibitors which exhibited IC_50_ values in the range of 13–75 μM. Among them, promising compound **A** (IC_50_ = 13.0 ± 1.5 µM) showed a competitive mode of inhibition^38^. The same authors also developed a new series of benzimidazole-1,2,3-triazole hybrid with IC_50_ values ranging from 25.2 to 176.5 μM. The most potent compound (Fig. 1 compound **B**) as a competitive inhibitor showed an IC_50_ = 25.2 ± 0.9 µM. Docking study demonstrated that phenoxy-1,2,3-triazole moiety was stable within the binding site through several π-π, π-cation, and hydrophobic interactions^39^. Recently, in a study carried out by our group, new hydrazineylideneindolinone derivatives linked to different phenoxymethyl-1,2,3-triazole were designed and the most potent compound (compound **C**) disclosed 46-fold improvement in the inhibitory activity compared to acarbose with an IC_50_ value of 750.0 µM. Docking evaluation exhibited H-bonding and π-alkyl interactions between phenoxy ring and Ala284. Also, the 1,2,3-triazole moiety recorded two π-alkyl interactions with leu283 and Ala555 as well as a π-sulfur interaction with Asp282^16^. As a result, phenoxy-1,2,3-triazole scaffold possessing a 3-benzyl substituent seems to be a good pharmacophore for the inhibition of α-glucosidase and can be further explored to design novel antidiabetic agents.

According to our limited literature review, no entry was found for cyanoacetohydrazide moiety as an α-glucosidase inhibitor. Our preliminary docking assessment disclosed that it is a valuable candidate for the exploration of the lead molecule. As depicted in Fig. 1, cyanoacetohydrazide effectively interacted with the critical binding site residues including Trp481, Asp518, Met519, Arg600 and can be considered as an ideal and novel fragment against α-glucosidase.

Pharmacophoric hybridization is known as one of the most efficient strategies in designing novel α-glucosidase inhibitors with improved affinity and efficacy. As a result, the benzyl-1,2,3-triazole moiety which seems to participate in π-stacking and hydrophobic interactions with the enzyme, was linked to the cyanoacetohydrazide pharmacophore. In vitro enzyme inhibition and the mechanism of action as well as docking studies were executed to determine plausible protein–ligand interactions.

### Chemistry

**Figure 2:**
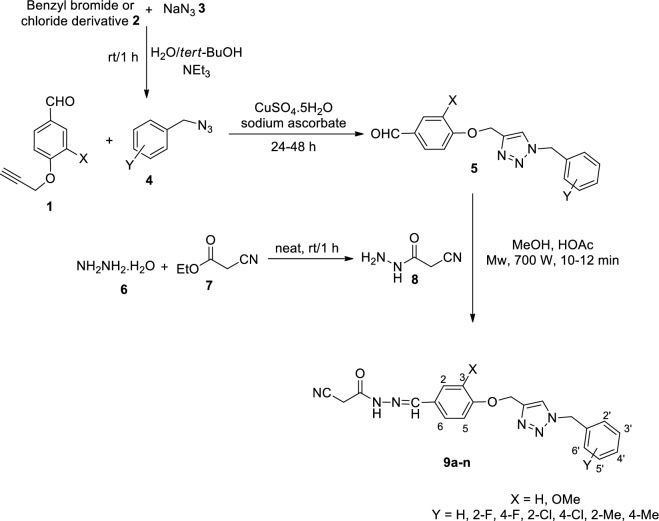
Synthesis of compounds **9a–n**.

Synthesis of the target compounds **9a**–**n** was schematically described in Fig. 2.

The corresponding derivatives were prepared by the reaction of 1,2,3-triazole-methoxy-benzaldehyde **5** and 2-cyanoacetohydrazide **8** in methanol in the presence of a few drops of acetic acid (HOAc) under microwave irradiation at 700 W for 10–12 min. Aldehyde **5** was prepared by the click reaction of compound **1** and in situ prepared azide derivatives **4** in the presence of triethylamine (NEt_3_), CuSO_4_.5H_2_O, and sodium ascorbate in H_2_O/*tert*-BuOH for 24–48 h. It should be mentioned that aldehyde **1** was prepared by the reaction of 4-hydroxy benzaldehyde or 4-hydroxy-3-methoxybenzaldehyde and propargyl bromide in DMF at 80 °C for 4–5 h^36^. Compound **8** was also obtained by the reaction of excess amount of hydrazine hydrate **6** and ethyl 2-cyanoacetate **7** at room temperature^40^.

All synthesized compounds were characterized by FTIR, ^1^H-NMR, ^13^C-NMR, elemental analysis, and HPLC (Supplementary Information). It should be noted that ^1^H and ^13^CNMR spectra of most compounds indicated the presence of two isomers probably due to restricted C-N amide bond rotation^41^. Also, the presence of two isomers was obvious in HPLC chromatograms.

### In vitro α-glucosidase inhibition

Fourteen cyanoacetohydrazide linked to 1,2,3-triazoles **9a–n** were synthesized (Table 1). They exhibited varying degrees of α-glucosidase inhibition with IC_50_ values in the range of 1.00 ± 0.01 to > 750 μM when compared with the standard inhibitor (acarbose: IC_50_ = 754.1 ± 0.5 μM).

**Table 1:** α-Glucosidase inhibitory activity of compounds **9a–n**.


Entry	Compound **9**	X	Y	IC_50_ (μM)^a^
1	**9a**	OMe	H	> 750
2	**9b**	OMe	2-F	1.50 ± 0.01
3	**9c**	OMe	4-F	9.73 ± 0.30
4	**9d**	OMe	2-Cl	13.97 ± 0.80
5	**9e**	OMe	4-Cl	1.00 ± 0.01
6	**9f**	OMe	2-Me	28.00 ± 0.10
7	**9g**	OMe	4-Me	22.80 ± 0.60
8	**9h**	H	H	271.17 ± 0.30
9	**9i**	H	2-F	45.89 ± 0.10
10	**9j**	H	4-F	56.64 ± 0.70
11	**9k**	H	2-Cl	74.68 ± 2.80
12	**9l**	H	4-Cl	21.66 ± 0.12
13	**9m**	H	2-Me	11.28 ± 0.20
14	**9n**	H	4-Me	82.36 ± 1.30
Acarbose				754.1 ± 0.5

^a^Data represented in terms of mean ± SD.

**Figure 3:**
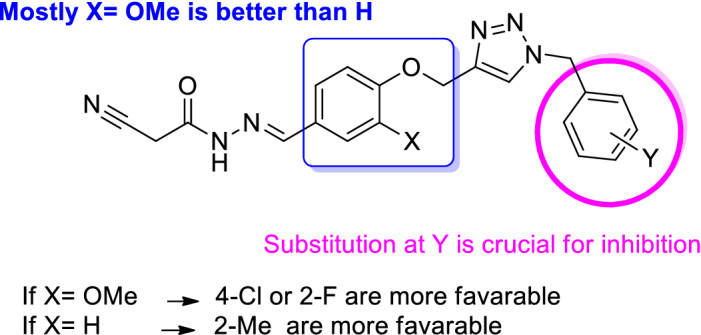
Summary of SAR studies of the library against α-glucosidase.

To explain the structure and observed activity correlations, cyanoacetohydrazide-1,2,3-triazole hybrids were divided into three categories based on the presence of methoxy group at X- position (**9a–g**), the unsubstituted group at X-position (**9h–n**) along with the substituents at the Y position of benzyl moiety to extract structure–activity relationships (SARs) of α-glucosidase inhibition.

(I) Among the **9a–g** bearing OMe at X- position, compound **9e** with 4-Cl substituent on the benzyl ring showed the most potent inhibitory activity (IC_50_ = 1.00 ± 0.01 µM) among all the synthesized compounds. It is worth mentioning that the most active compound **9e** recorded 754-fold better potency than the standard drug acarbose (IC_50_ 1.0 *Vs* 754.1 μM). Changing the chlorine position from *para* to *ortho* (**9d**) led to the decrease of inhibitory activity with an IC_50_ value of 13.97 ± 0.80 µM. Compound **9b** as the second most active analog (Y: 2-F, IC_50_ = 1.50 μM), showed similar activity compared to the most potent derivatives, **9e** (Y: 4-Cl, IC_50_ = 1.00 μM). Replacing halogen groups with methyl as an electron-donating group in **9f** (IC_50_ = 28.00 μM) and **9g** (IC_50_ = 22.80 μM) caused to decrease of inhibitory activity. Noteworthy, the removal of any substitution from Y position (compounds **9a** IC_50_ > 750 μM) resulted in considerable deterioration of the activity. Overall, it was understood that any substitution at the Y- position improved the inhibitory activity. Also, the electron-donating substituent is less effective compared to electron-withdrawing groups. The presence of halogen groups (2-F and 4-Cl) might play a key role in this inhibition of enzyme due to the high electronegativity, which makes the whole molecule more polar, and the enzyme might have better interaction with it.
(II) Similar to the previous set, among derivatives **9h–n**, any substitutions at the Y- position improved the activity significantly as compared with the unsubstituted analog. This trend can easily be seen in compound **9h** (Y = H) *vs* **9i** (Y = 2-F), **9j** (Y = 4-F), **9k** (Y = 2-Cl), **9l** (Y = 4-Cl), **9m** (Y = 2-Me), **9n** (Y = 4-Me). The activity of analogs containing electron-withdrawing group demonstrated that 4-Cl (**9l**) moiety at Y had good inhibition with IC_50_ value of 21.66 μM followed by 2-F (**9i,** IC_50_ = 45.89 μM) > 4-F (**9j,** IC_50_ = 56.64 μM) > 2-Cl (**9k,** IC_50_ = 74.68 μM). The minor difference in the activity of the last three analogs may be due to the difference in the position and electron-withdrawing power of the substituents on the benzyl moiety.
  By comparing the IC_50_ values in this set, it can be implied that *ortho*-methyl group as electron-donating substituent caused a significant improvement in the α-glucosidase inhibition with an IC_50_ value of 11.28 μM.
(III) Comparison of derivatives bearing the same substitution group at Y while X varies revealed that **9h** as an unsubstituted derivative at Y exhibited better potency compared to the **9a** counterpart. However, this trend was not followed in the rest of the derivatives as **9i**, **9j**, **9k**, **9l**, and **9n** were not more potent than their counterparts **9b**, **9c**, **9d**, **9l,** and **9g**. It can be understood that the SAR was mainly affected by the difference in substituents (Fig. 3).

Overall, it was perceived that any substitution at the Y position is favorable. Among the first set of compounds bearing OMe at X, it can be found that 4-Cl and 2-F substituents on the benzyl moiety played a substantial role in the anti-α-glucosidase activity. Although the presence of 2-CH_3_ at the Y-position had destructive effect on the first category, this derivative showed the highest activity in the second category.

To correlate the activity of present molecules with the previously published reports, different interesting SARs were obtained. The comparison of IC_50_ values of phenoxy derivatives with their corresponding methoxyphenyl analogs of biscoumarin derivatives (Compound **A**, Fig. 1) revealed that phenoxy analogs of biscoumarin (with 2-chloro and 4-nitro substituents) were more active than 4-methoxyphenoxy counterparts^38^. These results were supported in other studies on hydrazineylideneindolinone derivatives (Compound **C**, Fig. 1) so that phenoxy derivatives were more potent than methoxyphenoxy compounds^16^. Noteworthy, unlike the previous studies, in this work phenoxymethyl-1,2,3-triazole derivatives were more potent inhibitors than phenoxy-1,2,3-triazole counterparts.

Comparison of the benzyl substitutions showed that 2-fluorobenzyl of hydrazineylideneindolinone linked to phenoxymethyl-1,2,3-triazole derivatives (Compound **C**, Fig. 1) induced better α-glucosidase inhibitory activity than other derivatives^16^. Also, the same trend was observed by Xie et al., so the 2-fluorobenzyl moiety of isatin-thiazole scaffold disclosed better potency in comparison to different derivatives^42^. These results are in line with the current study. However, assessments on biscoumarin-1,2,3-triazole hybrids exhibited that 2-Cl substitution on the benzyl pendant recorded better potency than the rest of the derivatives^38^.

### Enzyme kinetic studies

**Figure 4:**
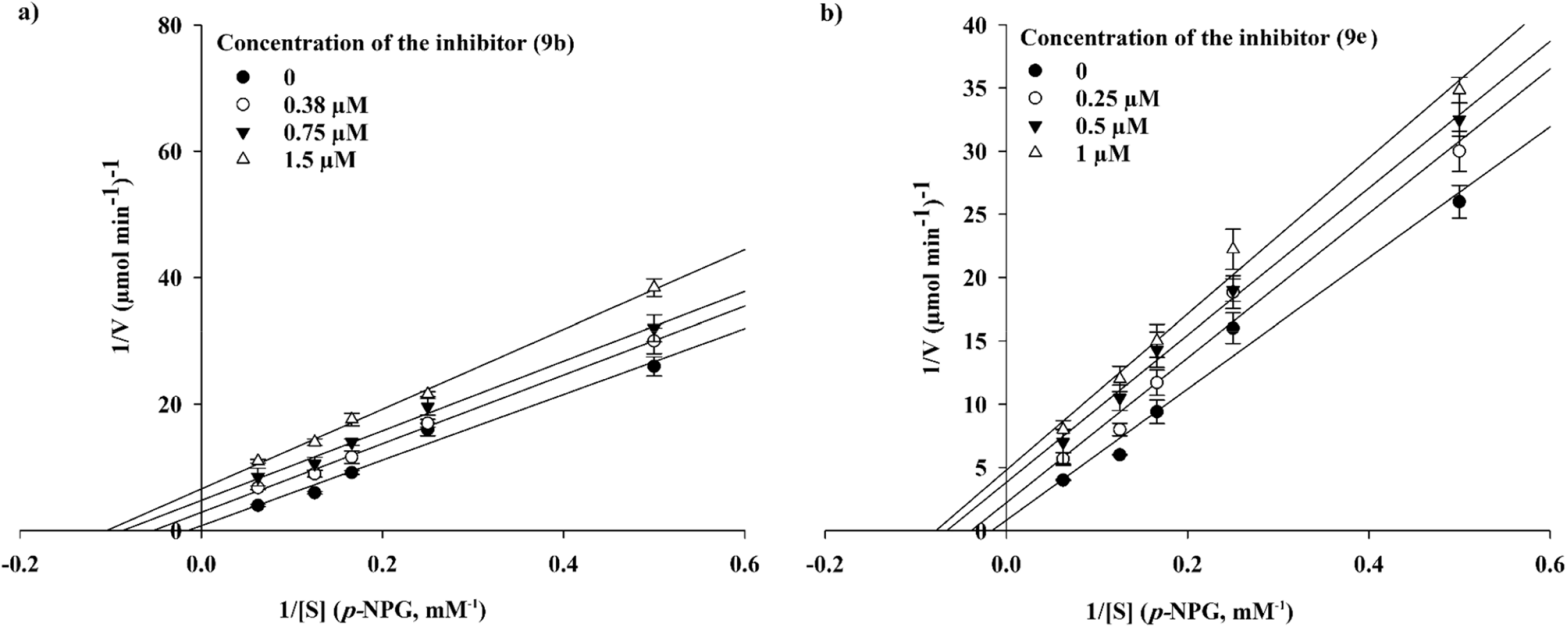
Kinetic study of α-glucosidase inhibition by compounds **9b** and **9e**. The Lineweaver–Burk plots were obtained in the absence and presence of different concentrations of inhibitors.

**Figure 5:**
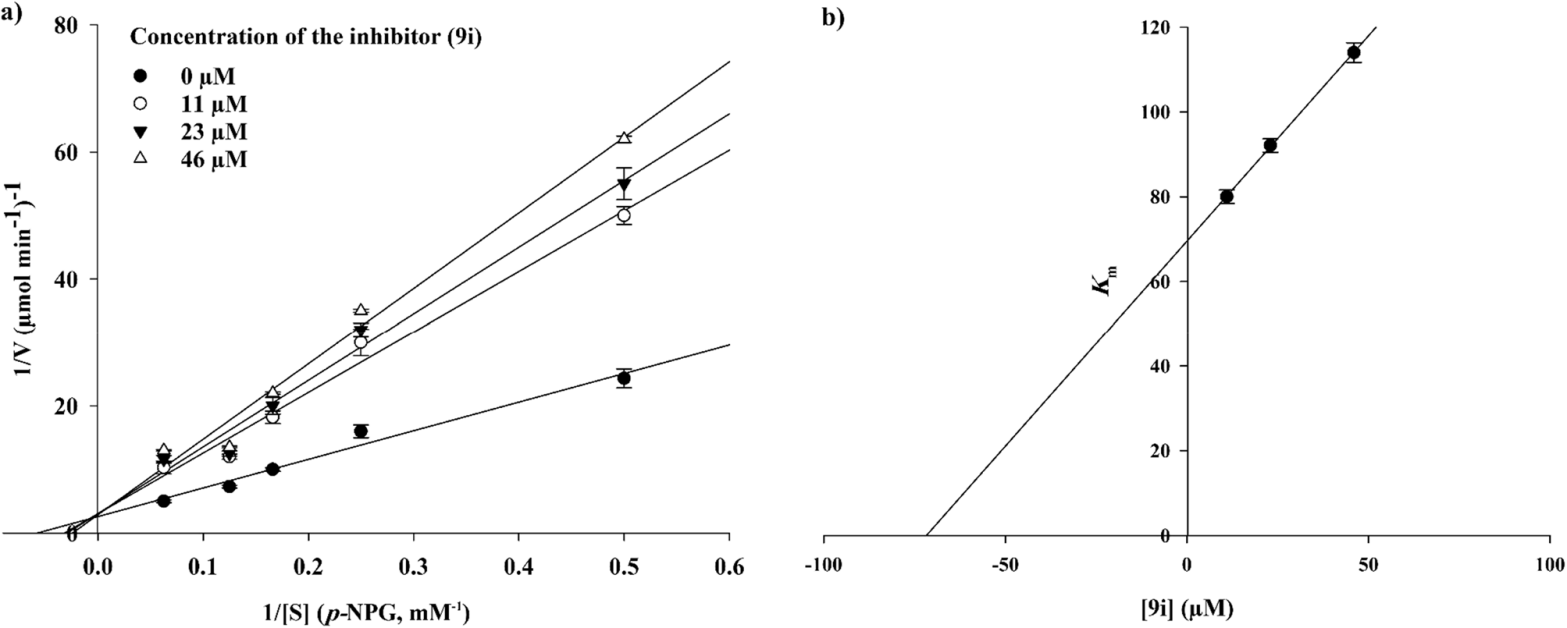
Kinetic study of α-glucosidase inhibition by compounds **9i**.

**Figure 6:**
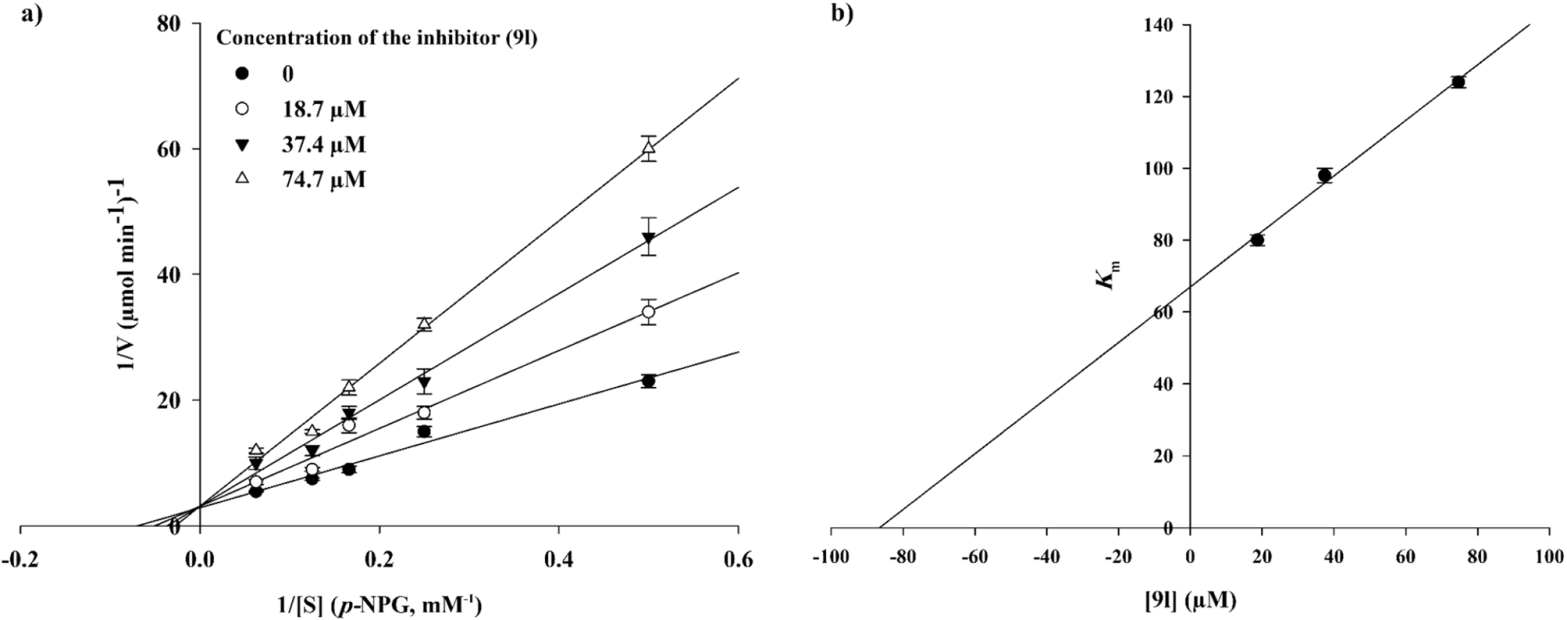
Kinetic study of α-glucosidase inhibition by compounds **9l**.

Kinetic studies were conducted for compounds **9b**, **9e**, **9i,** and **9l** to identify the type of inhibition. According to Fig. 4, the Lineweaver–Burk plot showed that the *K*_m_ and *V*_*max*_ gradually decreased with increasing the inhibitor concentration, indicating an uncompetitive inhibition for compounds **9b** and **9e** with *K*_*i*_ = 0.43 and 0.24 µM, respectively. However, investigation of their compartments **9i** and **9l** demonstrated different manner of α-glucosidase inhibition. As can be seen in Figs. 5 and 6, they revealed a competitive inhibition. The *K*_i_ value for compound **9i** was calculated as 75.0 µM and the corresponding value for compound **9l** was obtained as 85.0 µM.

### Fluorescence spectroscopy measurements

**Figure 7:**
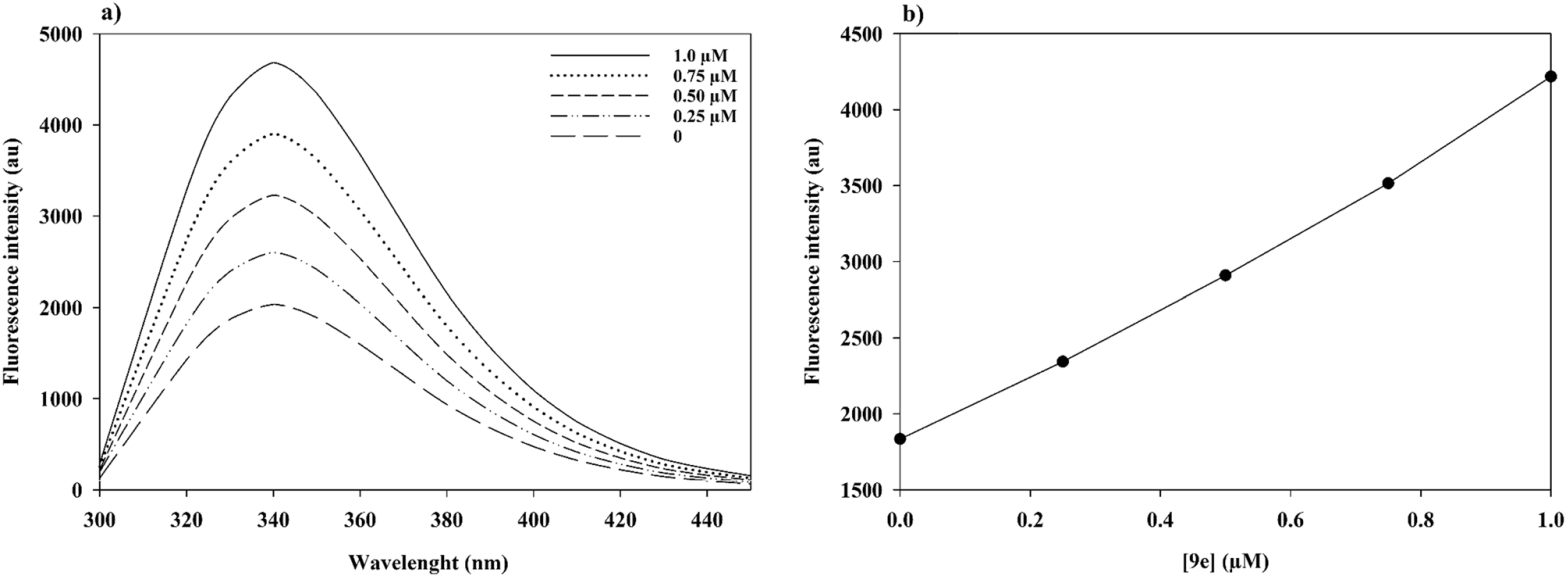
(Left) Fluorescence spectroscopy of α-glucosidase in the presence of different concentrations of compound **9e** (0–1.0 µM) in phosphate buffer (50 mM, pH 6.8). (Right) Inset shows the change in absorbance at 37 °C as a function of compound **9e**.

The intrinsic fluorescence property of α-glucosidase is generally due to the presence of tryptophan, tyrosine, and phenylalanine amino acids. α-Glucosidase has 18 tryptophan residues that eight are exposed to the solvent, and four are found in the proposed active site pocket (Trp381, Trp710, Trp715, and Trp789). Therefore, the conformation of the enzyme affected by the local tryptophan environment, can be followed by the change of fluorescence intensity^43,44^. In fact, fluorescence spectroscopy measurements could be used to predict the tertiary structure of the enzyme. To demonstrate the effect of compound **9e** on α-glucosidase activity, fluorescence spectra of the enzyme in the presence of various concentrations of **9e** were recorded (Fig. 7). As can be seen in Fig. 7, no shift was observed in the emission maximum (λ_max_ 340 nm) but a significant increase in fluorescence intensity was detected. This effect was directly dependent on the concentration of **9e** in the range of 0–1.0 μM.

### Thermodynamic analysis of binding of compound 9e to α-glucosidase

**Figure 8:**
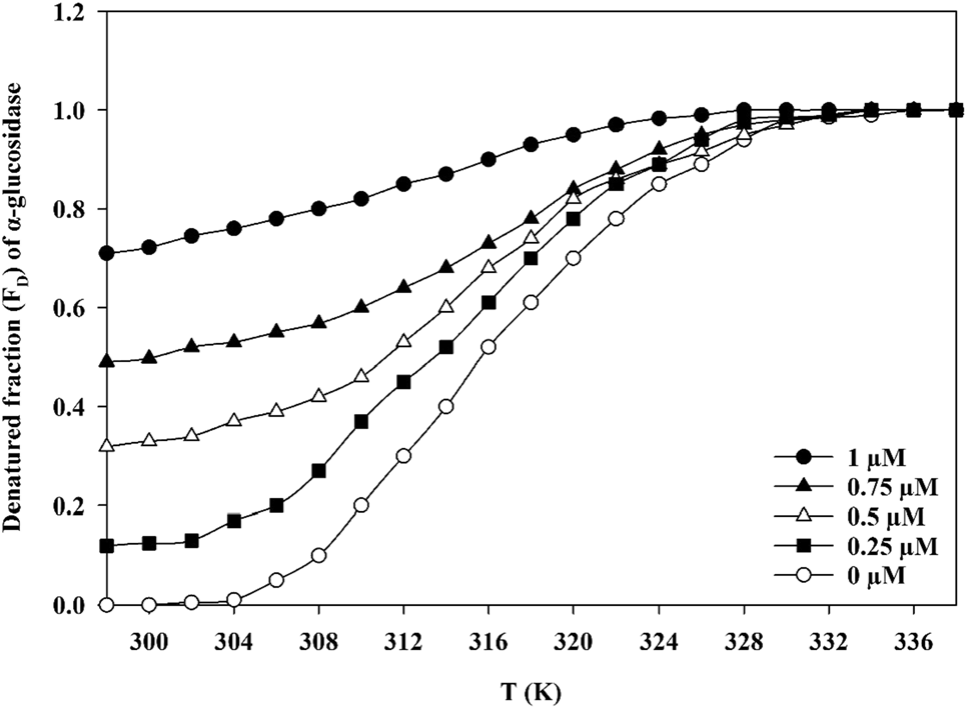
Fraction of unfolded α-glucosidase in various concentrations of compound **9e** in phosphate buffer (50 mM, pH 6.8).

Noncovalent interactions including hydrogen bonding, hydrophobic, electrostatic, and van der Waals forces are common forces between ligand and protein. To get insight into binding forces in the **9e**—α-glucosidase complex, the thermodynamic study was conducted and the thermodynamic parameters of the noncovalent interactions, i.e., standard enthalpy change (Δ*H*°), standard entropy change (Δ*S*°), and standard free energy change (Δ*G*°) were calculated. For this purpose, the stability of α-glucosidase in the presence or absence of compound **9e** was investigated by screening the fluorescence intensity at 340 nm at different temperatures (298–338 K) based on the equilibrium model (Native state ↔ Unfolded state). The start and end temperature points were 298 and 338 K, respectively. Denaturation profiles of α-glucosidase were then obtained by thermal scanning in the presence of various concentration of **9e**. As shown in Fig. 8, a sigmoidal curve observed by each profile indicated a single denaturant-dependent step based on the two-state theory.

The values of Δ*H*°_m_ and Δ*S*°_*m*_ were calculated as reported in Table 2. *T*_m_ was estimated to be the lowest for α-glucosidase that incubated in the presence of compound **9e** at the concentration of 1.0 µM (311 K), but in the case of concentrations of 0.5 and 0 µM, *T*_m_ was estimated to be 315 and 317 K, respectively. These results revealed that the most instability occurred at the higher concentration of compound **9e**.

**Table 2:** *T*_m_, Δ*H*_m_°, and Δ*S*_m_° values for α-glucosidase at variable concentrations of compound **9e**.

Concentration of compound **9e** (µM)	*T*_m_ (K)	Δ*H*_m_*°* (kJ mol^−1^)	Δ*S*_m_*°* (J mol^−1^ K^−1^)
0	317	5.66	17.85
0.25	316	94.47	298.95
0.50	315	112.86	358.28
0.75	313	177.09	565.76
1.0	311	597.50	1921.22

The forces between the protein and ligand can be categorized into *I*: Δ*H*° > 0, Δ*S*° > 0 for hydrophobic interactions; *II*: Δ*H*° < 0, Δ*S*° < 0 for van der Waals forces; *III*: Δ*H*° < 0, ΔS° < 0 for hydrogen bond and van der Waals interactions and *IV*: Δ*H*° < 0, Δ*S*° > 0 for electrostatic interactions; as non-covalent interactions. According to our results (Table 2), the presence of compound **9e** in aqueous solutions of α-glucosidase indicated the formation of hydrophobic interactions between nonpolar amino acid residues and the enzyme, confirming the unfolded state of the protein.

### Docking studies

Molecular docking studies were performed for compounds **9b** and **9e** to investigate the mode of their interactions with α-glucosidase (PDB ID: 5NN8) using the maestro molecular modeling platform of Schrödinger package. First to validate the in-silico procedure, the acarbose as a crystallographic inhibitor was docked into human lysosomal acid-α-glucosidase. The superimposed structure of acarbose and its crystallographic conformation recorded an RMSD value of 1.69 Å. Next, the docking assessments of the compounds were done based on the same protocol performed on the crystallographic inhibitor.

**Figure 9:**
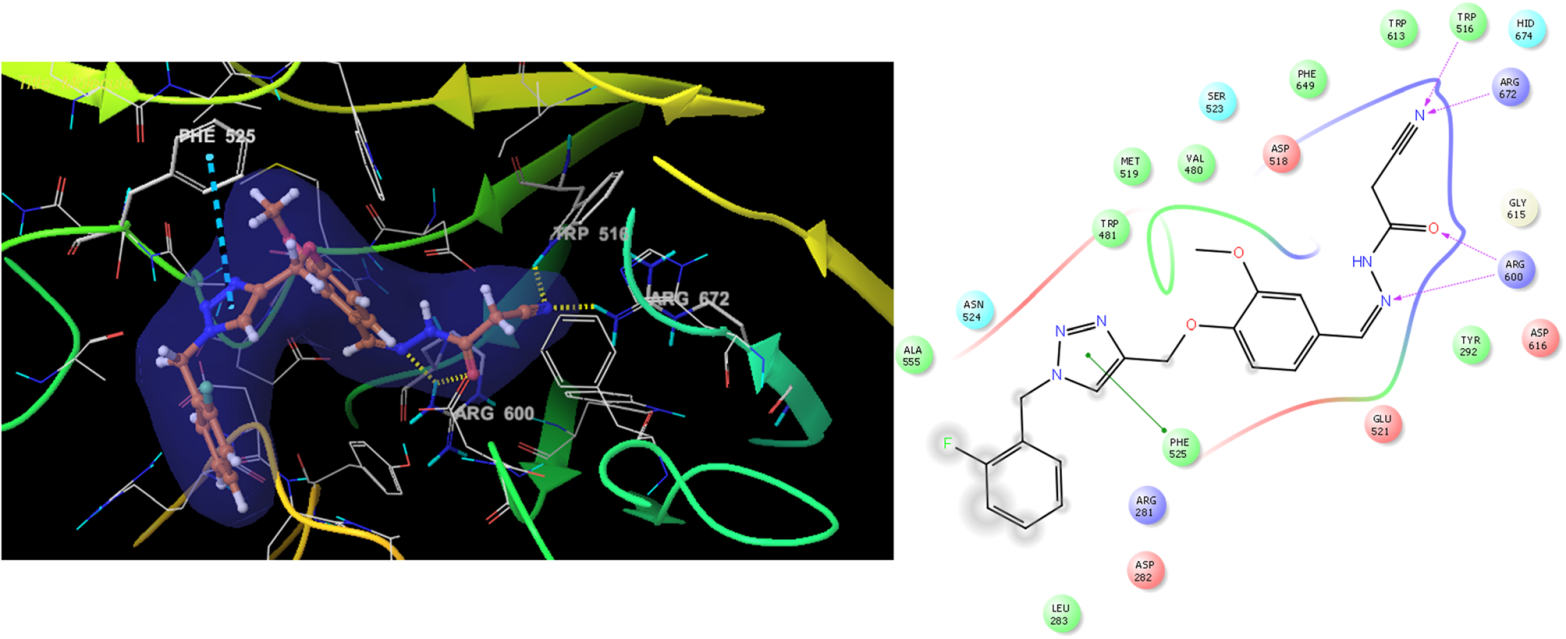
3D and 2D diagram of compound **9b** within the binding pocket of α-glucosidase and potential distribution surface diagram.

Figure 9 presented the binding pattern of derivative **9b** with the binding site of α-glucosidase (glide score = -− 7.04 kcal/mol). Derivative **9b** oriented within the α-glucosidase active site so that phenoxy-cyanoacetohydrazide penetrated the deep gorge of the binding site and the substituted moiety oriented toward the entrance of the active site. In detail, the nitrogen of cyanoacetohydrazide pendant was fixed between the Trp616 (essential residue) and Arg672. Carbonyl and hydrazine moieties of cyanoacetohydrazide group also participated in H-bound interactions with Arg600 (essential residue). The *ortho-*fluorobenzyl ring was stacked with Phe525 thus stabilizing the molecule at the entrance of the active site to get the suppressed conformation of α-glucosidase.

**Figure 10:**
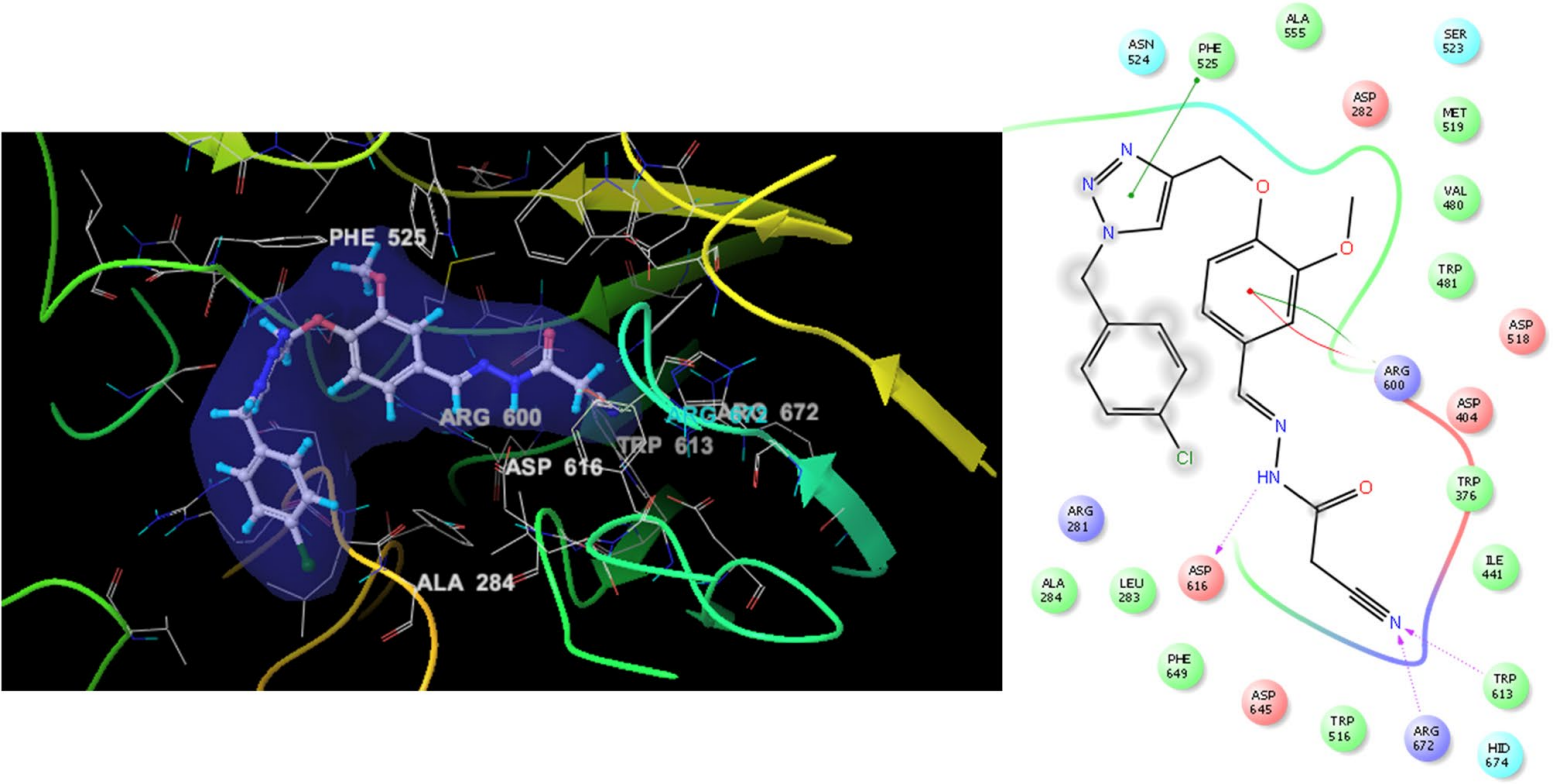
3D and 2D diagram of compound **9e** within the binding pocket of α-glucosidase and potential distribution surface diagram.

According to molecular docking study, **9e** recorded the glide score of − 6.89 kcal/mol. As shown in Fig. 10, phenoxy-cyanoacetohydrazide oriented toward the inner core of the binding pocket, while the *para-*chlorobenzyl part (substituted moiety) of the compound bonded near the active site entrance. Focusing on the cyanoacetohydrazide pendant, confirmed our designing strategy so that nitrogen of the cyano group exhibited two H-bonding interactions with Trp613 (essential residue) and Arg672. Also, NH of hydrazide participated in H-bonding interaction with Asp616 (essential residue). There were π–π stacking and π–cation interactions between Arg600 (essential residue) and the phenoxy linker. Also, 1,2,3-triazole ring recorded a π–π stacking interaction with Phe525.

### ADME-toxicity profiles and physicochemical properties

The pkCSM server^45^ was used to predict the ADME-toxicity properties of synthesized compounds. As shown in Table 3, all derivatives showed good human intestinal absorption, low clearance values, and low toxicity.

**Table 3:** ADMET prediction of the synthesized derivatives as α-glucosidase inhibitors.

	Absorption	Distribution	Metabolism							Excretion	Toxicity
	Human intestinal absorption (% absorbed)	VDss (logL/Kg)	2D6	3A4	1A2	2C19	2C9	2D6	3A4	Total clearance (log mL/min/kg)	Oral rat acute toxicity (mol/kg)
			Substrate		Inhibitor						
**9a**	98.657	− 0.401	No	Yes	No	Yes	Yes	No	Yes	**0.649**	**2.259**
**9b**	100	− 0.544	No	Yes	No	Yes	No	No	Yes	0.545	2.317
**9c**	96.769	− 0.537	No	Yes	No	Yes	No	No	Yes	0.478	2.31
**9d**	99.833	− 0.381	No	Yes	No	Yes	Yes	No	Yes	0.216	2.364
**9e**	93.892	− 0.371	No	Yes	No	Yes	Yes	No	Yes	0.151	2.356
**9f**	99.125	− 0.346	No	Yes	No	Yes	Yes	No	Yes	0.662	2.311
**9g**	93.184	− 0.336	No	Yes	No	Yes	Yes	No	Yes	0.652	2.304
**9h**	94.348	− 0.303	No	Yes	Yes	Yes	Yes	No	Yes	0.698	2.191
**9i**	**100**	− 0.453	No	Yes	No	No	Yes	No	Yes	0.590	2.272
**9j**	100	− 0.453	No	Yes	No	No	Yes	No	Yes	0.523	2.272
**9k**	95.524	− 0.282	No	Yes	No	Yes	Yes	No	Yes	0.020	2.3
**9l**	95.524	− 0.282	No	Yes	No	Yes	Yes	No	Yes	− 0.044	2.3
**9m**	94.816	− 0.248	No	Yes	No	Yes	Yes	No	Yes	0.711	2.243
**9n**	94.816	− 0.248	No	Yes	No	Yes	Yes	No	Yes	0.701	2.243

Significant values are in bold.

The results of drug-likeness properties were shown in Table 4. All compounds exhibited appropriate molecular properties with no drug-likeness rules violations^46^.

**Table 4:** Drug-likeness properties of synthesized compounds.

Compound	M_W_	Num. rotatable bonds	Num. H-bond acceptors	Num. H-bond donors	Log *P*
**9a**	404.43	9	8	1	2.2777
**9b**	422.42	9	8	1	2.41688
**9c**	422.42	9	8	1	2.41688
**9d**	438.875	9	8	1	2.93118
**9e**	438.875	9	8	1	2.93118
**9f**	418.457	9	8	1	2.5862
**9g**	418.457	9	8	1	2.5862
**9h**	374.404	8	7	1	2.26918
**9i**	392.394	8	7	1	2.40828
**9j**	392.394	8	7	1	2.40828
**9k**	408.849	8	7	1	2.92258
**9l**	408.849	8	7	1	2.92258
**9m**	388.431	8	7	1	2.5776
**9n**	388.431	8	7	1	2.5776

## Conclusion

Novel cyanoacetohydrazide linked to 1,2,3-triazoles were designed, synthesized, and characterized via spectroscopic techniques and evaluated for their α-glucosidase inhibitory potential. These compounds except **9a** demonstrated considerable inhibitory activity against α-glucosidase with IC_50_ value of 1.0 to 271.17 µM compared to acarbose as the positive control (IC_50_ value of 754.1 µM). Compound **9e** (IC_50_ = 1.00 ± 0.01 μM) with having *para* chlorobenzyl ring and **9b** (IC_50_ = 1.50 ± 0.01 μM) bearing *ortho* fluorobenzyl pendant group were found to be the most potent α-glucosidase inhibitors. Kinetic studies revealed that they **9b** and **9e** behaved uncompetitively against the enzyme with *K*_*i*_ = 0.43 and 0.24 μM, respectively. Also, the binding affinity between compound **9e** at different concentrations and α-glucosidase was recorded using fluorescence measurements. It indicated the inhibition of α-glucosidase due to conformational changes of the enzyme. According to the thermodynamic studies, hydrophobic interactions were found to be responsible for the formation of **9e**—α-glucosidase complex. The in-silico studies confirmed the designing strategy so that cyanoacetohydrazide group was able to form several important interactions within the cavity which supported the high potency of these compounds and phenoxy-1,2,3-triazole moiety stabilized the derivatives through several hydrophobic and hydrophilic interactions. Interestingly substituted moiety at Y position occupied the entrance of the active site to get the suppressed conformation of α-glucosidase. These results were in accordance with enzymatic assessments that any substitution at the Y position was favorable. As expected, developed pharmacophores used in the design of these hybrids, are involved in the interactions with the enzyme.

## Materials and methods

All chemicals and reagents were purchased from Merck and Aldrich. Melting points were determined using Kofler hot stage apparatus and are uncorrected. The IR spectra were obtained on a Nicolet Magna FTIR 550 spectrometer (potassium bromide disks). NMR spectra were recorded on a Varian-INOVA 500 MHz and chemical shifts were expressed as δ (ppm) with tetramethylsilane as internal standard. Analytical HPLC evaluation was performed on a YL9100 HPLC system (Korea) equipped with UV detectors using a RP column (Teknokroma, C18, 5 μm, 150 × 4.6 mm) and solvent: methanol (solvent A) and water, a gradient of 0–100% solvent A in 11 min, 1 min at 0%, to 50% within 3 min, to 100% at 6 min, to 0 within 5 min (total run time 11 min); flow rate, 1 mL/min; detection, 254 nm; injection volume, 20 μL.

### Synthesis of compounds 9

The click reaction was conducted by a mixture of aldehyde **1** and in situ prepared azide derivative **4** to obtain compound **5**^16^. For this purpose, benzyl chloride/bromide derivative **2** (1.1 mmol) and sodium azide **3** (0.06 g, 0.9 mmol) in the presence of triethylamine (0.13 g, 1.3 mmol) in the mixture of water (4 mL) and *tert*-butyl alcohol (4 mL) was stirred at room temperature for 30 min. Next, compound **1** (0.5 mmol) and CuSO_4_·5H_2_O (7 mol%) were added to the reaction mixture and it was continued for 24–48 h. After completion of the reaction (checked by TLC), the mixture was poured on crushed ice, the precipitates were filtered off and washed with water. Compound **5** was used for further steps with no purification. A mixture of compound **5** (1 mmol) and 2-cyanoacetohydrazide **8** (1 mmol) in methanol (8 mL), in the presence of a few drops of HOAc was irritated under microwave irradiation at 700 W for 10–12 min (1 min interval). After completion of the reaction (checked by TLC), the mixture was poured on crushed ice, the precipitates were filtered off and washed frequently with water (Supplementary Information).

#### N′-(4-((1-benzyl-1H-1,2,3-triazol-4-yl)methoxy)-3-methoxybenzylidene)-2-cyanoacetohydrazide (9a)

Deep yellow precipitates, Yield: 92%, mp 142–144 °C, IR (KBr, cm^−1^): 3415, 3222, 2924, 2250, 1690, 1616, 1586. ^1^H-NMR (500 MHz, DMSO-*d*_6_) (two isomers): 11.71 (s, 1H, NH), 11.63 (s, 1H, NH), 8.33 (s, 1H, triazole), 8.31 (s, 1H, triazole), 7.96 (s, 1H, CH), 7.93 (s, 1H, CH), 7.40–7.32 (m, 7H, H5, H6, H2′, H3′, H4′, H5′, H6′), 7.21 (s, 1H, H2), 7.18 (s, 1H, H2), 5.62 (s, 2H, CH_2_), 5.26 (s, 2H, CH_2_), 5.17 (s, 2H, CH_2_), 4.21 (s, 2H, CH_2_), 3.79 (s, 3H, OMe), 3.77 (s, 3H, OMe) ppm. ^13^C-NMR (125 MHz, DMSO-*d*_6_) (two isomers): 165.1, 153.3, 149.8, 149.7, 144.8, 136.4, 130.4, 129.2, 128.7, 128.5, 127.5, 121.7, 116.6, 115.3, 113.6, 113.1, 110.2, 109.4, 62.1, 55.9, 53.4, 46.3, 24.8 ppm. Calcd for C_21_H_20_N_6_O_3_: C, 62.37; H, 4.98; N, 20.78. Found: C, 62.50; H, 5.18; N, 20.51.

#### 2-Cyano-N′-(4-((1-(2-fluorobenzyl)-1H-1,2,3-triazol-4-yl)methoxy)-3-methoxybenzylidene)acetohydrazide (9b)

Pale yellow precipitates, Yield: 74%, mp 124–126 °C, 3416, 3227, 2936, 2250, 1692, 1600. ^1^H-NMR (500 MHz, DMSO-*d*_*6*_) (two isomers): 11.70 (s, 1H, NH), 11.60 (s, 1H, NH), 8.27 (s, 1H, triazole), 8.08 (s, 1H, triazole), 7.92 (s, 1H, CH), 7.43–7.18 (m, 14H, H2, H5, H6, H3′, H4′, H5′, H6′), 5.68 (s, 2H, CH_2_), 5.16 (s, 2H, CH_2_), 4.21 (s, 2H, CH_2_), 3.77 (s, 3H, OMe) ppm. ^13^C-NMR (125 MHz, DMSO-*d*_6_): 165.1, 160.6 (d, *J*_*C-F*_ = 245.4 Hz), 149.8, 149.7, 144.8, 131.3 (d, *J*_*C-F*_ = 3.8 Hz), 131.2, 127.5, 125.6, 125.3 (d, *J*_*C-F*_ = 3.7 Hz), 123.3., 123.2, 121.7, 116.7, 116.1 (d, *J*_*C-F*_ = 20.6 Hz), 113.6, 109.4, 62.0, 55.9, 47.4, 24.8 ppm. Calcd for C_21_H_19_FN_6_O_3_: C, 59.71; H, 4.53; N, 19.90. Found: C, 59.62; H, 4.39; N, 19.78.

#### 2-Cyano-N′-(4-((1-(4-fluorobenzyl)-1H-1,2,3-triazol-4-yl)methoxy)-3-methoxybenzylidene)acetohydrazide (9c)

Pale yellow precipitates, Yield: 68%, mp 131–133 °C, IR (KBr, cm^−1^): 3416, 2963, 2850, 2250, 1690, 1603. ^1^H-NMR (500 MHz, DMSO-*d*_6_) (two isomers): 11.71 (s, 1H, NH), 11.62 (s, 1H, NH), 8.30 (s, 1H, triazole), 8.10 (s, 1H, triazole), 7.93 (s, 1H, CH), 7.88 (s, 1H, CH), 7.42–7.34 (m, 3H, H6, H2′, H6′), 7.21 (t, *J* = 8.8 Hz, 2H, H3′, H5′), 7.20–7.18 (m, 2H, H2, H5), 5.61 (s, 2H, CH_2_), 5.16 (s, 2H, CH_2_), 4.22 (s, 2H, CH_2_), 3.79 (s, 3H, OMe), 3.77 (s, 3H, OMe) ppm. ^13^C-NMR (125 MHz, DMSO-*d*_6_): 165.1, 162.4 (d, *J*_*C-F*_ = 243.3 Hz), 150.0, 149.8, 149.7, 132.7, 130.8 (d, *J*_*C-F*_ = 8.4 Hz), 127.5, 125.4, 122.3, 121.7, 116.6, 116.1 (d, *J*_*C-F*_ = 21.5 Hz), 113.6, 109.4, 62.1, 55.9, 52.5, 24.8 ppm Calcd for C_21_H_19_FN_6_O_3_: C, 59.71; H, 4.53; N, 19.90. Found: C, 59.58; H, 4.36; N, 20.21.

#### N′-(4-((1-(2-chlorobenzyl)-1H-1,2,3-triazol-4-yl)methoxy)-3-methoxybenzylidene)-2-cyanoacetohydrazide (9d)

Yellow precipitates, Yield: 86%, mp 148–150 °C, IR (KBr, cm^−1^): 3415, 3224, 2922, 2250, 1698, 1662, 1613. ^1^H-NMR (500 MHz, DMSO-*d*_*6*_) (two isomers): 11.71 (s, 1H, NH), 11.61 (s, 1H, NH), 8.27 (s, 1H, triazole), 8.09 (s, 1H, triazole), 7.92 (s, 1H, CH), 7.88 (s, 1H, CH), 7.52 (d, *J* = 7.7 Hz, 1H, H3′), 7.40–7.34 (m, 3H, H6, H5′, H6′), 7.24–7.18 (m, 3H, H2, H5, H4′), 5.72 (s, 2H, CH_2_), 5.17 (s, 2H, CH_2_), 4.21 (s, 2H, CH_2_), 3.77 (s, 3H, OMe), 3.67 (s, 3H, OMe) ppm. ^13^C-NMR (125 MHz, DMSO-*d*_*6*_) (two isomers): 165.1, 151.1, 149.8, 149.7, 149.6, 144.7, 133.7, 133.1, 131.0, 130.7, 130.1, 128.2, 127.5, 125.9, 125.8, 121.7, 116.6, 113.7, 109.4, 62.0, 55.9, 51.1, 24.8 ppm. Calcd for C_21_H_19_ClN_6_O_3_: C, 57.47; H, 4.36; N, 19.15. Found: C, 57.60; H, 4.54; N, 18.92.

#### N′-(4-((1-(4-chlorobenzyl)-1H-1,2,3-triazol-4-yl)methoxy)-3-methoxybenzylidene)-2-cyanoacetohydrazide (9e)

Creamy precipitates, Yield: 87%, mp 199–201 °C, IR (KBr, cm^−1^): 3417, 2928, 2250, 1683, 1616. ^1^H-NMR (500 MHz, DMSO-*d*_*6*_) (two isomers): 11.71 (s, 1H, NH), 11.63 (s, 1H, NH), 8.34 (s, 1H, triazole), 8.08 (s, 1H, triazole), 7.92 (s, 1H, CH), 7.88 (s, 1H, CH), 7.44 (d, *J* = 8.2 Hz, 2H, H3′, H5′), 7.35–7.33 (m, 3H, H6, H2′, H6′), 7.20–7.17 (m, 2H, H2, H5), 5.62 (s, 2H, CH_2_), 5.16 (s, 2H, CH_2_), 4.21 (s, 2H, CH_2_), 3.79 (s, 3H, OMe), 3.77 (s, 3H, OMe) ppm. ^13^C-NMR (125 MHz, DMSO-*d*_*6*_): 165.1, 151.9, 149.8, 149.7, 144.7, 135.4, 133.4, 130.4, 129.2, 127.5, 125.5, 121.7, 116.6, 113.7, 109.4, 62.1, 55.9, 52.5, 24.8 ppm. Calcd for C_21_H_19_ClN_6_O_3_: C, 57.47; H, 4.36; N, 19.15. Found: C, 57.21; H, 4.20; N, 19.31.

#### 2-Cyano-N′-(3-methoxy-4-((1-(2-methylbenzyl)-1H-1,2,3-triazol-4-yl)methoxy)benzylidene)acetohydrazide (9f)

Pale yellow precipitates, Yield: 94%, mp 105–107 °C, IR (KBr, cm^−1^): 3415, 2960, 2250, 1688, 1602, 1578. ^1^H-NMR (500 MHz, DMSO-*d*_*6*_) (two isomers): 11.71 (s, 1H, NH), 11.62 (s, 1H, NH), 8.20 (s, 1H, triazole), 8.10 (s, 1H, triazole), 7.93 (s, 1H, CH), 7.90 (s, 1H, CH), 7.34–7.18 (m, 12H, H2, H5, H6, H3′, H4′, H5′), 7.09 (d, *J* = 7.6 Hz, 1H, H6′), 5.63 (s, 2H, CH_2_), 5.17 (s, 2H, CH_2_), 4.22 (s, 2H, CH_2_), 3.79 (s, 3H, OMe), 3.77 (s, 3H, OMe), 2.35 (s, 3H, Me) ppm. ^13^C-NMR (125 MHz, DMSO-*d*_*6*_):165.1, 149.8, 149.7, 144.8, 136.8, 134.5, 113.3, 130.9, 129.1, 128.8, 127.5, 126.7, 125.6, 121.7, 116.6, 113.7, 109.5, 62.1, 55.9, 51.4, 24.8, 19.1 ppm. Calcd for C_22_H_22_N_6_O_3_: C, 63.15; H, 5.30; N, 20.08. Found: C, 63.37; H, 5.44; N, 19.79.

#### 2-Cyano-N′-(3-methoxy-4-((1-(4-methylbenzyl)-1H-1,2,3-triazol-4-yl)methoxy)benzylidene)acetohydrazide (9g)

Off white precipitates, Yield: 84%, mp 98–100 °C, IR (KBr, cm^−1^): 3415, 3222, 2923, 2250, 1683, 1601. ^1^H NMR (500 MHz, DMSO-*d*_*6*_) (two isomers): 11.71 (s, 1H, NH), 11.61 (s, 1H, NH), 8.26 (s, 1H, triazole), 8.04 (s, 1H, triazole), 7.92 (s, 1H, CH), 7.88 (s, 1H, CH), 7.33–7.17 (m, 7H, H2, H5, H6, H2′, H3′, H5′, H6′), 5.55 (s, 2H, CH_2_), 5.15 (s, 2H, CH_2_), 4.21 (s, 2H, CH_2_), 3.78 (s, 3H, OMe), 3.77 (s, 3H, OM), 2.28 (s, 3H, Me), 2.24 (s, 3H, Me) ppm. ^13^C-NMR (125 MHz, DMSO-*d*_6_): 165.1, 151.5, 149.8, 149.7, 144.8, 138.0, 133.4, 129.8, 128.5, 127.5, 125.3, 121.7, 116.6, 113.6, 109.4, 62.1, 55.9, 53.1, 24.8, 21.1 ppm. Calcd for C_22_H_22_N_6_O_3_: C, 63.15; H, 5.30; N, 20.08. Found: C, 63.43; H, 5.18; N, 19.88.

#### N′-(4-((1-benzyl-1H-1,2,3-triazol-4-yl)methoxy)benzylidene)-2-cyanoacetohydrazide (9h)

Off white precipitates, Yield: 65%, mp 121–123 °C, IR (KBr, cm^−1^): 3425, 3222, 3100, 22,250, 1680, 1606. ^1^H NMR (500 MHz, DMSO-*d*_6_) (two isomers): 11.68 (s, 1H, NH), 11.58 (s, 1H, NH), 8.31 (s, 1H, triazole), 8.10 (s, 1H, triazole), 7.95 (s, 1H, CH), 7.90 (s, 1H, CH), 7.64 (d, *J* = 8.4 Hz, 2H, H2, H6), 7.53 (d, *J* = 8.5 Hz, 2H, H2, H6), 7.39–7.31 (m, 7H, H2′, H3′, H4′, H5′, H6′), 7.09 (d, *J* = 8.4 Hz, 2H, H3, H5), 5.61 (s, 2H, CH_2_), 5.19 (s, 2H, CH_2_), 4.18 (s, 2H, CH_2_), 4.15 (s, 2H, CH_2_) ppm. ^13^C NMR (125 MHz, DMSO-*d*_6_) (two isomers): 165.0, 160.1, 144.6, 143.3, 136.4, 129.3, 129.2, 129.1, 128.6, 128.4, 127.2, 125.2, 116.6, 115.5, 61.7, 53.3, 24.7 ppm. Calcd for C_20_H_18_N_6_O_2_: C, 64.16; H, 4.85; N, 22.45. Found: C, 64.35; H, 4.63; N, 22.60.

#### 2-Cyano-N′-(4-((1-(2-fluorobenzyl)-1H-1,2,3-triazol-4-yl)methoxy)benzylidene)acetohydrazide (9i)

Off white precipitates, Yield: 60%, mp 123–125 °C, IR (KBr, cm^−1^): 3415, 2963, 2850, 2250, 1681, 1607. ^1^H NMR (500 MHz, DMSO-*d*_6_) (two isomers): 11.68 (s, 1H, NH), 11.60 (s, 1H, NH), 8.30 (s, 1H, triazole), 8.12 (s, 1H, triazole), 7.96 (s, 1H, CH), 7.90 (s, 1H, CH), 7.65 (d, *J* = 8.5 Hz, 2H, H2, H6), 7.55–7.53 (m, 1H, H4′), 7.44–7.41 (m, 1H, H3′), 7.38–7.35 (m, 2H, H3′, H4′), 7.29–7.22 (m, 4H, 2 × H5′, 2 × H6′), 7.10 (d, *J* = 8.5 Hz, 2H, H3, H5), 5.69 (s, 2H, CH_2_), 5.20 (s, 2H, CH_2_), 4.19 (s, 2H, CH_2_), 4.16 (s, 2H, CH_2_) ppm. ^13^C NMR (125 MHz, DMSO-*d*_6_) (two isomers): 165.0, 160.6 (d, *J*_*C-F*_ = 245.6 Hz), 160.1, 159.1, 148.1, 144.6, 131.3, 131.2 (d, *J*_*C-F*_ = 10.8 Hz), 129.3, 129.1, 127.2, 125.4, 125.3 (d, *J*_*C-F*_ = 14.3 Hz), 123.2 (d, *J*_*C-F*_ = 14.8 Hz), 116.6, 116.1 (d, *J*_*C-F*_ = 20.9 Hz), 115.6, 115.5, 61.6, 47.4, 24.7 ppm. Calcd for C_20_H_17_FN_6_O_2_: C, 61.22; H, 4.37; N, 21.42. Found: C, 61.40; H, 4.21; N, 21.28.

#### 2-Cyano-N′-(4-((1-(4-fluorobenzyl)-1H-1,2,3-triazol-4-yl)methoxy)benzylidene)acetohydrazide (9j)

Off white precipitates, Yield: 93%, mp 108–110 °C, IR (KBr, cm^−1^): 3422, 2961, 2250, 1681, 1605. ^1^H NMR (500 MHz, DMSO-*d*_6_) (two isomers): 11.67 (s, 1H, NH), 11.59 (s, 1H, NH), 8.30 (s, 1H, triazole), 8.10 (s, 1H, triazole), 7.95 (s, 1H, CH), 7.89 (s, 1H, CH), 7.64 (d, *J* = 8.1 Hz, 2H, H2, H6), 7.53 (d, *J* = 8.6 Hz, 2H, H2, H6), 7.41–7.38 (m, 2H, H2′, H6′), 7.21 (t, *J* = 8.7 Hz, 2H, H3′, H5′), 7.08 (d, *J* = 8.1 Hz, 2H, H3, H5), 7.04 (d, *J* = 8.8 Hz, 2H, H3, H5), 5.61 (s, 2H, CH_2_), 5.19 (s, 2H, CH_2_), 4.17 (s, 2H, CH_2_), 4.15 (s, 2H, CH_2_) ppm. ^13^C NMR (125 MHz, DMSO-*d*_6_) (two isomers): 165.0, 162.4 (d, *J*_*C-F*_ = 243.3 Hz), 160.0, 148.0, 144.6, 132.7 (d, *J*_*C-F*_ = 2.9 Hz), 130.8, 130.7, 130.6, 130.5, 127.2, 125.2, 116.6, 116.1, 115.1 (d, *J*_*C-F*_ = 21.4 Hz), 61.7, 52.5, 25.2, 24.7 ppm. Calcd for C_20_H_17_FN_6_O_2_: C, 61.22; H, 4.37; N, 21.42. Found: C, 61.51; H, 4.14; N, 21.63.

#### N′-(4-((1-(2-chlorobenzyl)-1H-1,2,3-triazol-4-yl)methoxy)benzylidene)-2-cyanoacetohydrazide (9k)

White precipitates, Yield: 73%, mp 103–105 °C, IR (KBr, cm^−1^): 3415, 3217, 3075, 2925, 2250, 1677, 1607. ^1^H NMR (500 MHz, DMSO-*d*_6_) (two isomers): 11.67 (s, 1H, NH), 11.59 (s, 1H, NH), 8.28 (s, 1H, triazole), 8.10 (s, 1H, triazole), 7.95 (s, 1H, CH), 7.64 (d, *J* = 8.5 Hz, 2H, H2, H6), 7.52 (d, *J* = 7.7 Hz, 1H, H3′), 7.42–7.35 (m, 2H, H4′, H5′), 7.23 (d, *J* = 7.7 Hz, 1H, H6′), 7.09 (d, *J* = 8.5 Hz, 2H, H3, H5), 5.72 (s, 2H, CH_2_), 5.20 (s, 2H, CH_2_), 4.18 (s, 2H, CH_2_) ppm. ^13^C NMR (125 MHz, DMSO-*d*_6_) (two isomers): 165.0, 160.0, 144.6, 143.0, 133.7, 133.1, 131.0, 130.7, 130.1, 129.3, 129.1, 128.2, 127.2, 125.7, 116.6, 115.6, 115.5, 61.6, 51.1, 24.7 ppm. Calcd for C_20_H_17_ClN_6_O_2_: C, 58.75; H, 4.19; N, 20.56. Found: C, 58.93; H, 3.90; N, 20.38.

#### N′-(4-((1-(4-chlorobenzyl)-1H-1,2,3-triazol-4-yl)methoxy)benzylidene)-2-cyanoacetohydrazide (9l)

Off white precipitates, Yield: 89%, mp 123–125 °C, IR (KBr, cm^−1^): 3414, 3235, 2924, 2250, 1682, 1638. ^1^H NMR (500 MHz, DMSO-*d*_6_) (two isomers): 11.67 (s, 1H, NH), 11.59 (s, 1H, NH), 8.31 (s, 1H, triazole), 8.11 (s, 1H, triazole), 7.95 (s, 1H, CH), 7.89 (s, 1H, CH), 7.64 (d, *J* = 8.1 Hz, 2H, H2, H6), 7.53 (d, *J* = 8.6 Hz, 2H, H2, H6), 7.44 (d, *J* = 8.0 Hz, 2H, H3′, H5′), 7.34 (d, *J* = 8.0 Hz, 2H, H2′, H6′), 7.28 (d, *J* = 8.0 Hz, 2H, H2′, H6′), 7.08 (d, *J* = 8.1 Hz, 2H, H3, H5), 6.98 (d, *J* = 6.5 Hz, 2H, H3, H5), 5.62 (s, 2H, CH_2_), 5.19 (s, 2H, CH_2_), 4.17 (s, 2H, CH_2_), 4.15 (s, 2H, CH_2_) ppm. ^13^C NMR (125 MHz, DMSO-*d*_6_) (two isomers): 165.0, 160.0, 144.6, 135.4, 133.4, 130.4, 130.1, 129.4, 129.3, 129.2, 129.1, 127.2, 125.3, 116.6, 115.5, 61.6, 52.5, 24.7 ppm. Calcd for C_20_H_17_ClN_6_O_2_: C, 58.75; H, 4.19; N, 20.56. Found: C, 58.60; H, 4.37; N, 20.70.

#### 2-Cyano-N′-(4-((1-(2-methylbenzyl)-1H-1,2,3-triazol-4-yl)methoxy)benzylidene)acetohydrazide (9m)

White precipitates, Yield: 490%, mp 116–118 °C, IR (KBr, cm^−1^): 3415, 3222, 2960, 2250, 1682, 1605. ^1^H NMR (500 MHz, DMSO-*d*_6_) (two isomers): 11.67 (s, 1H, NH), 11.60 (s, 1H, NH), 8.21 (s, 1H, triazole), 8.11 (s, 1H, triazole), 7.95 (s, 1H, CH), 7.90 (s, 1H, CH), 7.26–7.08 (m, 8H, H2, H3, H5, H6, H3′, H4′, H5′, H6′), 5.62 (s, 2H, CH_2_), 5.20 (s, 2H, CH_2_), 4.17 (s, 2H, CH_2_), 2.30 (s, 3H, Me) ppm. ^13^C NMR (125 MHz, DMSO-*d*_6_) (two isomers): 165.0, 160.0, 144.6, 136.8, 134.5, 130.9, 129.3, 129.1, 129.0, 128.8, 127.1, 126.7, 125.5, 116.6, 115.6, 115.5, 61.6, 51.5, 24.7, 19.1 ppm. Calcd for C_21_H_20_N_6_O_2_: C, 64.94; H, 5.19; N, 21.64. Found: C, 65.15; H, 5.28; N, 21.44.

#### 2-Cyano-N′-(4-((1-(4-methylbenzyl)-1H-1,2,3-triazol-4-yl)methoxy)benzylidene)acetohydrazide (9n)

Off white precipitates, Yield: 84%, mp 128–130 °C, IR (KBr, cm^−1^): 3417, 2924, 2956, 2250, 1682, 1606. ^1^H NMR (500 MHz, DMSO-*d*_6_) (two isomers): 11.67 (s, 1H, NH), 11.58 (s, 1H, NH), 8.26 (s, 1H, triazole), 8.10 (s, 1H, triazole), 7.95 (s, 1H, CH), 7.89 (s, 1H, CH), 7.64 (d, *J* = 8.1 Hz, 2H, H2, H6), 7.54 (d, *J* = 8.7 Hz, 2H, H2, H6), 7.22 (d, *J* = 7.7 Hz, 2H, H2′, H6′), 7.17 (d, *J* = 7.7 Hz, 2H, H3′, H5′), 7.08 (d, *J* = 8.1 Hz, 2H, H3, H5), 7.03 (d, *J* = 7.5 Hz, 2H, H3, H5), 5.55 (s, 2H, CH_2_), 5.17 (s, 2H, CH_2_), 4.18 (s, 2H, CH_2_), 4.15 (s, 2H, CH_2_), 2.27 (s, 3H, Me), 2.23 (s, 3H, Me) ppm. ^13^C NMR (125 MHz, DMSO-*d*_6_) (two isomers): 165.0, 160.1, 145.1, 144.6, 138.4, 138.0, 133.4, 129.8, 129.3, 129.1, 128.5, 128.2, 127.2, 125.1, 116.6, 115.6, 115.5, 61.7, 53.1, 24.7, 21.1 ppm. Calcd for C_21_H_20_N_6_O_2_: C, 64.94; H, 5.19; N, 21.64. Found: C, 65.22; H, 5.31; N, 21.80.

### In vitro α-glucosidase inhibition assay

α-Glucosidase (*Saccharomyces cerevisiae*, EC3.2.1.20, 20 U/mg) and the substrate, *p*-nitrophenyl-β-D-glucopyranoside (*p*-NPG) were purchased from Sigma-Aldrich and the assay was performed exactly according to our previous report^14^. In this respect, various concentrations of each synthesized compound dissolved in DMSO, were added to potassium phosphate buffer (50 mM, pH 6.8) including enzyme (at final concentration of 0.1 U/mL), in a 96-well plate. After a 10-min incubation at 37 °C, *p*-NPG was added to each well to achieve final concentration of 4 mM. Then, the plate was re-incubated at 37 °C for 20 min. It should be noted that the final concentration of DMSO in each enzymatic solution was 10%. Finally, the change in the absorbance was measured at 405 nm using spectrophotometer (Synergy HTX Multi-Mode Microplate Reader–BioTek, Germany). Acarbose, the standard inhibitor of α-glucosidase was used as the positive control and the enzyme activity in the absence of each inhibitor was considered as the negative control. The percentage of inhibition for compounds and control was calculated using Eq. (1):

1 $${\text{Inhibition}}\% \, = \, \left[ {\left( {{\text{OD}}_{{\text{negative control}}} - {\text{ OD}}_{{{\text{sample}}}} } \right)/{\text{OD}}_{{\text{negative control}}} } \right] \, \times {1}00 \, \left( {{\text{OD }} = {\text{ optical density at 4}}0{\text{5 nm}}} \right).$$

IC_50_ values were calculated from the nonlinear regression curve using the Logit method.

### Enzyme kinetic studies

The mode of inhibition of compounds **9b**, **9e**, **9i**, and **9l** was investigated against α-glucosidase activity with different concentrations of *p*-nitrophenyl *α*-D-glucopyranoside (*p*-NPG) (2–16 mM) as the substrate in the absence and presence of those compounds at different concentrations (**9b**: 0, 0.38, 0.75, and 1.50 µM; **9e**: 0, 0.25, 0.50, and 1.00 µM; **9i**: 0, 11.00, 23.00, and 45.00 µM; **9l**: 0, 18.70, 37.40, and 74.40 µM). A Lineweaver–Burk plot was generated to identify the type of inhibition and the Michaelis–Menten constant.

### Fluorescence spectroscopy measurements

Compound **9e** at different concentrations (0–1.0 µM) was added into the 3 mL solution containing a fixed amount of α-glucosidase (0.1 U/mL). All mixtures were held for 10 min to equilibrate before measurements. Then, the fluorescence emission spectra were measured from 300 to 450 nm at the excitation wavelength of 280 nm on a Synergy HTX multi-mode reader (Biotek Instruments, Winooski, VT, USA) equipped with a 1.0 cm quartz cell holder. The fluorescence spectra of the buffer containing compound **9e** in the absence of the enzyme were subtracted as the background fluorescence^47^.

### Thermodynamic analysis against α-glucosidase

Thermodynamic analysis was performed as described by Mojtabavi et al., the fluorescent intensity data were plotted as a function of temperature, and the thermodynamic profile was computed^48,49^. Therefore, the denatured fraction (F_D_) of protein was calculated from Eq. (2), assuming a two-state mechanism for the protein denaturation:

2 $${\text{F}}_{{\text{D}}} = \, \left( {{\text{Y}}_{{\text{N}}} - {\text{Y}}_{{{\text{obs}}}} } \right)/\left( {{\text{Y}}_{{\text{N}}} - {\text{Y}}_{{\text{D}}} } \right).$$

In Eq. (2), Y_obs_, Y_N_, and Y_D_ are the observed absorbance, the values of absorbance characteristics of a fully native and denatured conformation, respectively. Equation (3) was used to calculate the apparent equilibrium constant (K) for a reversible denaturation process between native and denatured protein states:

3 $${\text{K}} = {\text{ F}}_{{\text{D}}} /{1}{-}{\text{F}}_{{\text{D}}} = \, \left( {{\text{Y}}_{{{\text{obs}}}} - {\text{Y}}_{{\text{D}}} } \right)/\left( {{\text{Y}}_{{\text{N}}} - {\text{Y}}_{{\text{D}}} } \right).$$

The standard Gibbs free energy change (Δ*G*°) for protein denaturation is given by the Eq. (4):

4 $$\Delta G^{^\circ } \, = G^{^\circ }_{{\text{D}}} - G^{^\circ }_{{\text{N}}} = \, {-}RT{\text{ln}}K,$$

where T and R are the absolute temperature and the universal gas constant, respectively. The Gibbs free energy (Δ*G*°) is the most valuable standard of protein conformational stability in thermal denaturation. The integrated Gibbs–Helmholtz equation was utilized for measuring changes in the Gibbs energy of a system as a function of temperature (Eq. (5)):

5 $$\Delta G^{^\circ } = \, \Delta H^{^\circ }_{{\text{m}}} \left( {{1}{-}\left( {T/T_{{\text{m}}} } \right)} \right) \, {-} \, \Delta C_{{\text{p}}} \left[ {\left( {T_{{\text{m}}} {-}T} \right) + T{\text{Ln }}\left( {T/T_{{\text{m}}} } \right)} \right],$$

where Δ*C*_p_ is the heat capacity of protein denaturation. The Δ*C*_p_ (11.6 kJ/mol K) of the α-glucosidase denaturation was taken from van der Kamp et al. report^48^. In thermal denaturation, *T*_m_ is the temperature at which the protein is half denatured. Δ*H*°_m_ and Δ*S*°_*m*_ are the standard enthalpy and entropy of denaturation. The standard entropy was calculated from a relation between the standard enthalpy (Δ*S*) and entropy (Δ*H*) of denaturation as bellow:

$$\Delta H = T_{{\text{m}}} \;\Delta S.$$

### Molecular docking

The molecular docking of compounds **9b** and **9e** was performed using the maestro molecular modeling platform (version 10.5), Schrödinger suites^50^. X-ray crystallographic structure of α-glucosidase in complex with acarbose (PDB ID: 5NN8) was obtained from www.rcsb.com^44^. A protein preparation wizard was used to remove water molecules and co-crystallized atoms from the protein and prepare the receptor. Moreover, heteroatom states were generated at pH: 7.4 by EPIK, and H-bonds were assigned using PROPKA at the same pH. 2D structure of ligands was drawn in Hyperchem and the energies were minimized using molecular mechanics and molecular quantum approaches. Next, the ligand preparation wizard was used to prepare the ligand using the OPLS_2005 force field^51^. Acarbose, compounds **9b** and **9e** were docked into the binding sites using glide tasked to report ten poses per ligand with flexible ligand sampling and extra precision^52^.

### In-silico pharmacokinetic properties of synthesized compounds

SwissADME (http://www.swissadme.ch/) and pkCSM (http://biosig.unimelb.edu.au/pkcsm/) servers were used to determine the physicochemical and drug-likeness properties of the derivatives.

## Supplementary Information

Supplementary Information.
