# Supplementary material for: Cyanoacetohydrazide linked to 1,2,3-triazole derivatives: a new class of α-glucosidase inhibitors
Source: Sci Rep. 2022 May 23;12:8647. doi: 10.1038/s41598-022-11771-y (PMC9125976; doi:10.1038/s41598-022-11771-y)

**Supplementary file**

**Cyanoacetohydrazide linked to 1,2,3-triazole derivatives: A new class of α-glucosidase inhibitors**

**NMR Spectra**

*N'-(4-((1-Benzyl-1H-1,2,3-triazol-4-yl)methoxy)-3-methoxybenzylidene)-2-cyanoacetohydrazide (****9a****)*

*
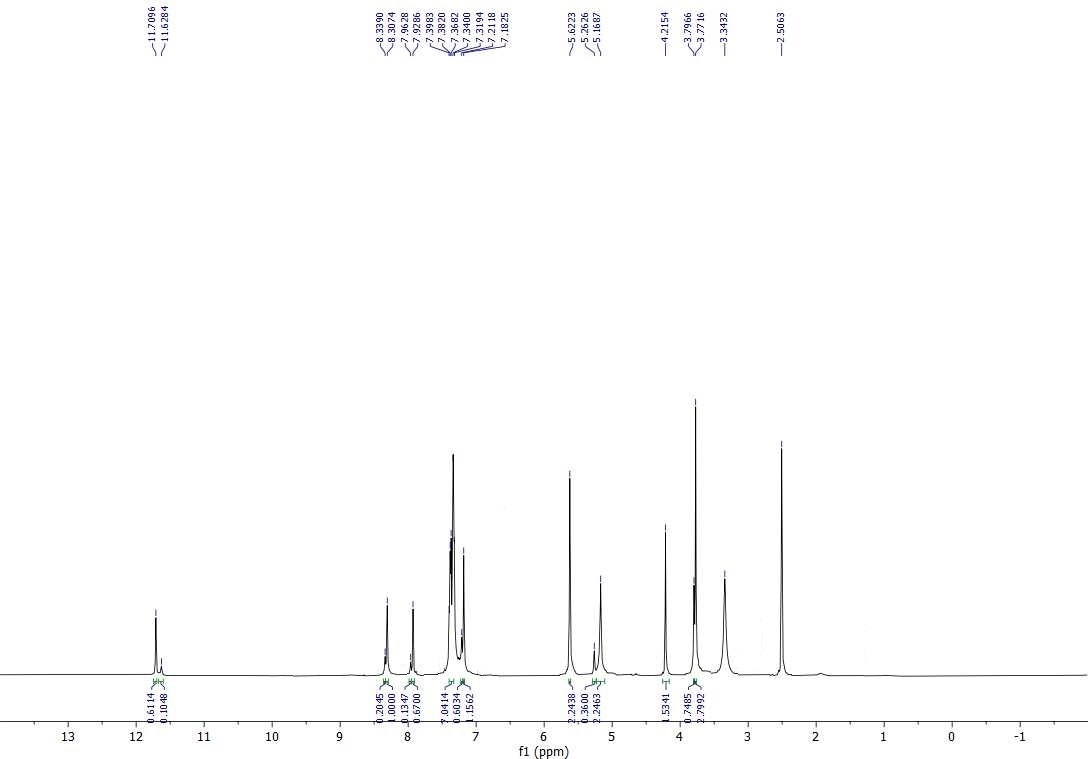
*


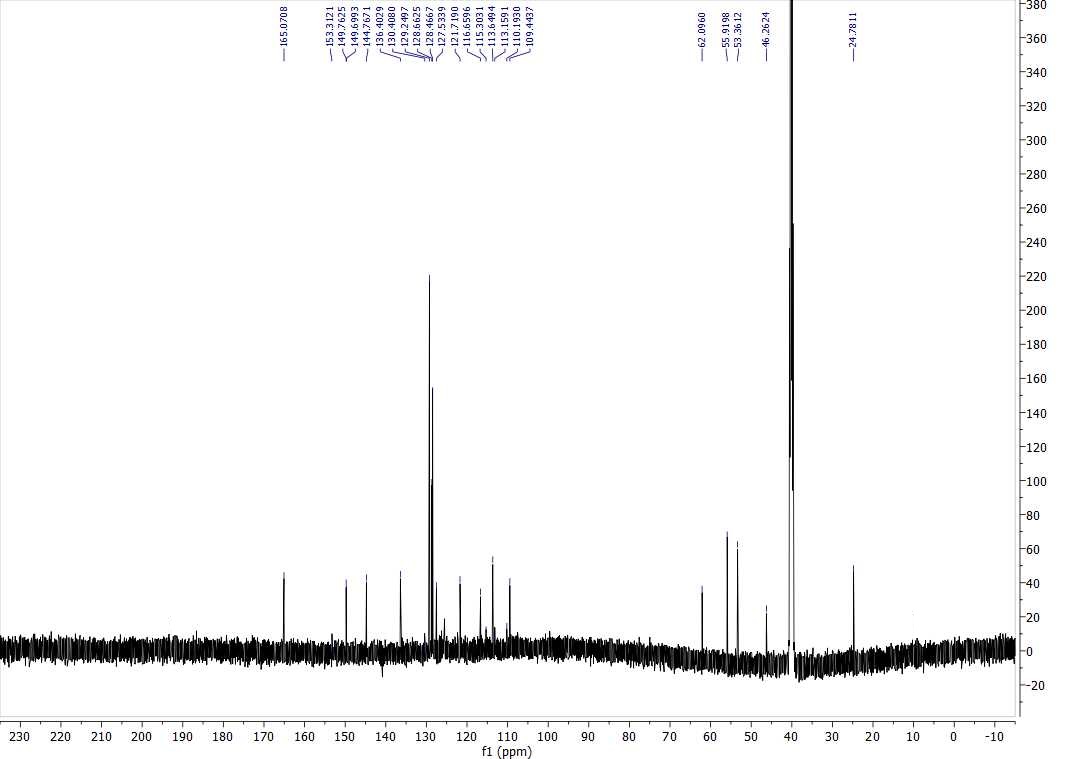


*2-Cyano-N'-(4-((1-(2-fluorobenzyl)-1H-1,2,3-triazol-4-yl)methoxy)-3-methoxybenzylidene)acetohydrazide (****9b****)*

*
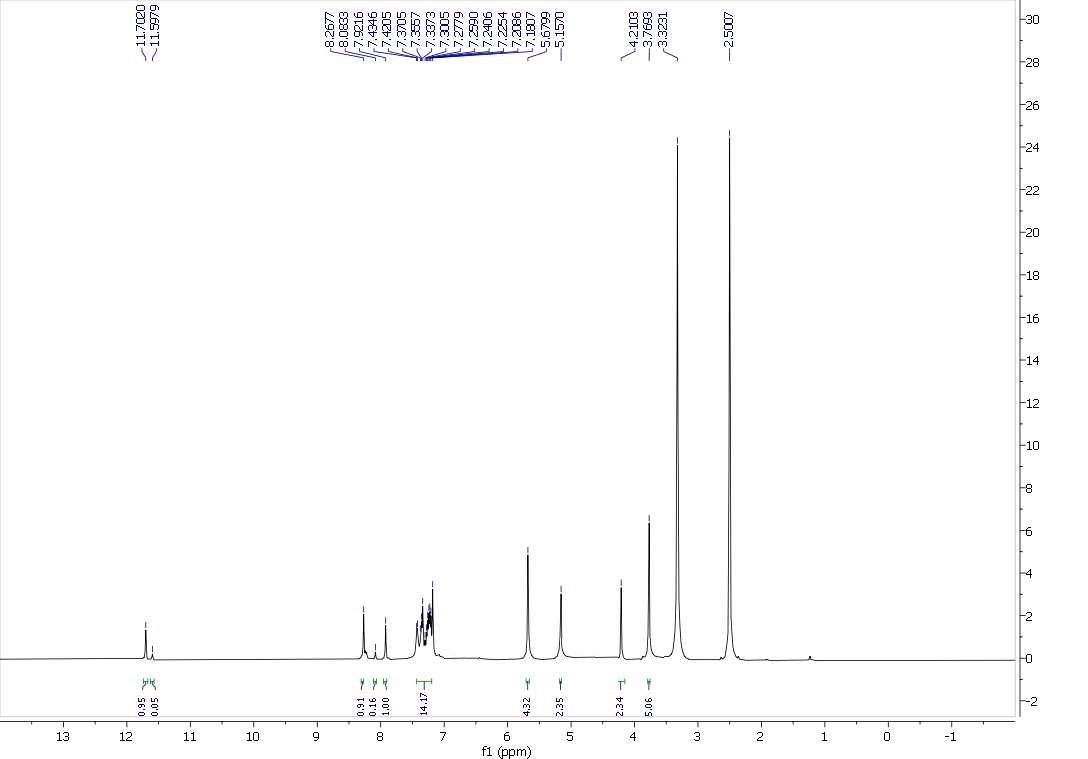
*

*
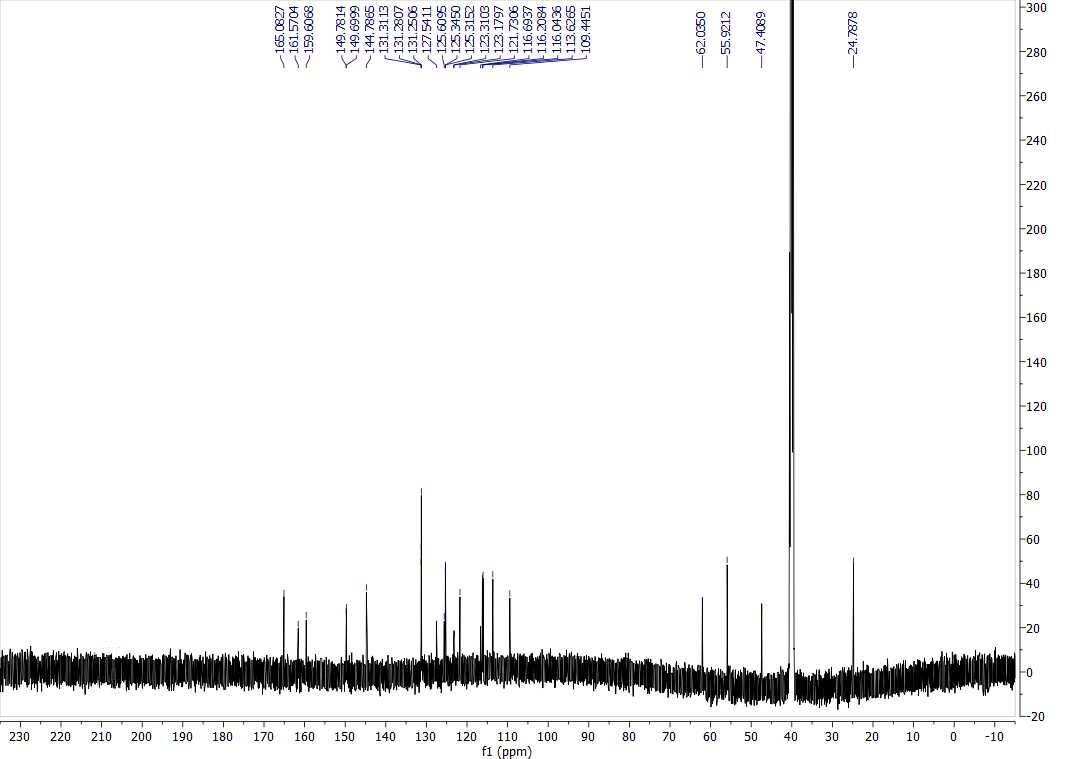
*

*2-Cyano-N'-(4-((1-(4-fluorobenzyl)-1H-1,2,3-triazol-4-yl)methoxy)-3-methoxybenzylidene)acetohydrazide (****9c****)*

*
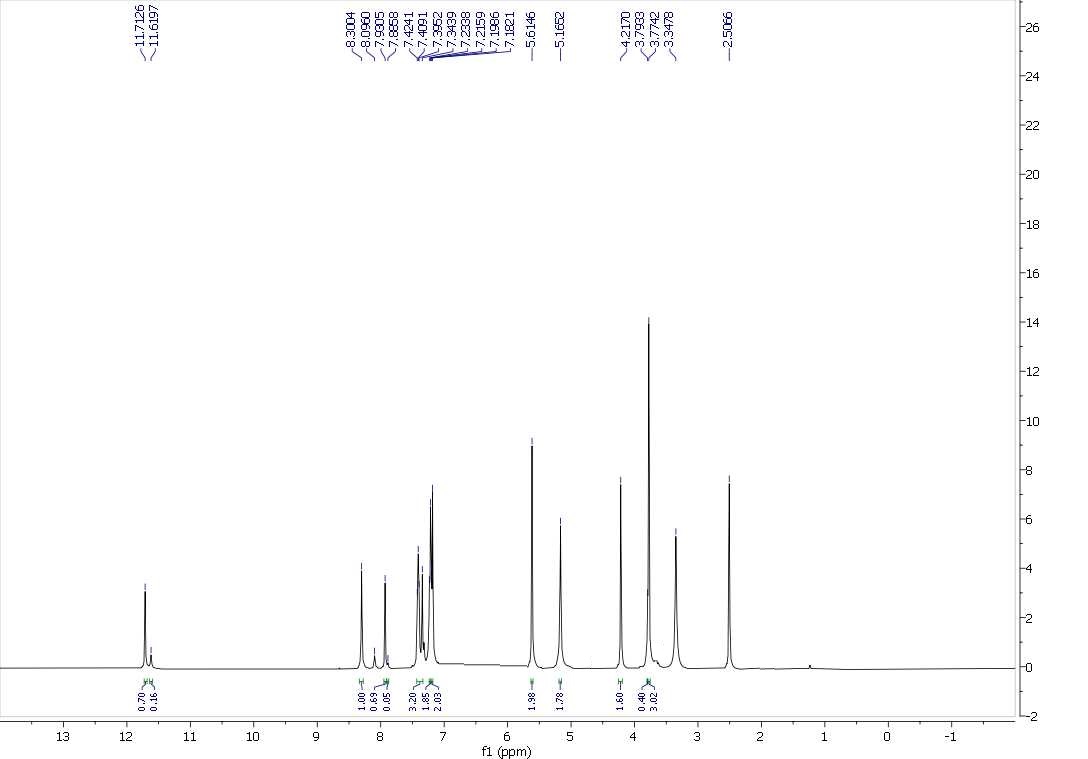
*


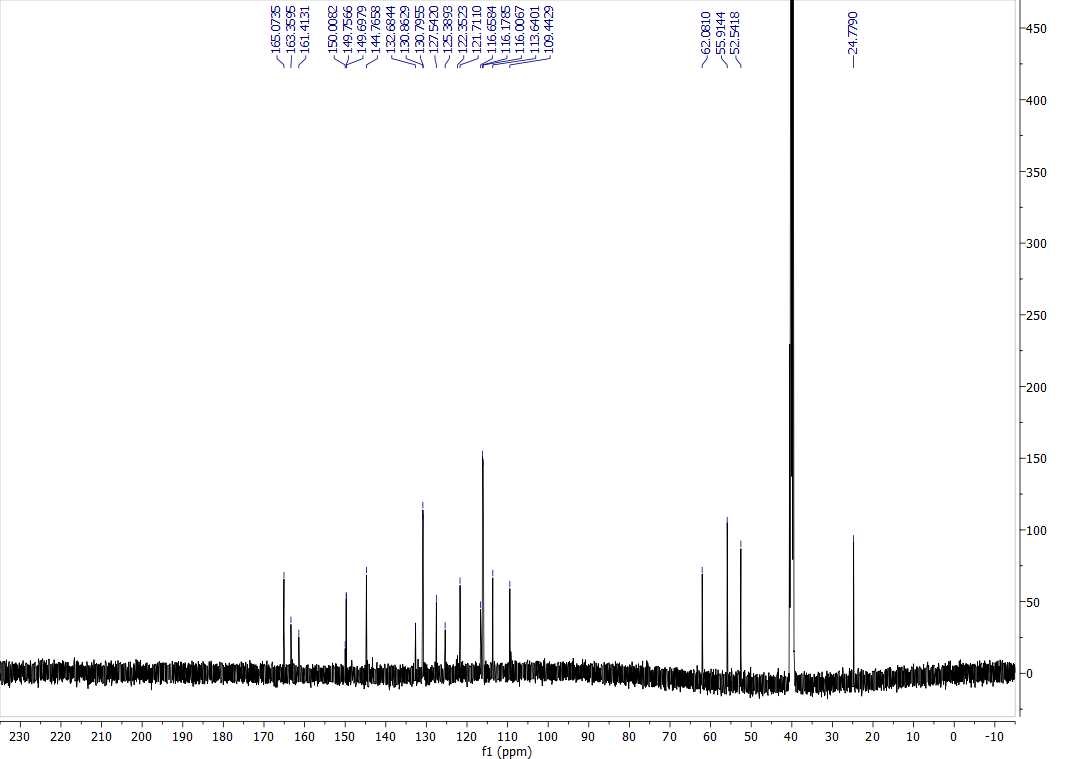


*N'-(4-((1-(2-Chlorobenzyl)-1H-1,2,3-triazol-4-yl)methoxy)-3-methoxybenzylidene)-2-cyanoacetohydrazide (****9d****)*


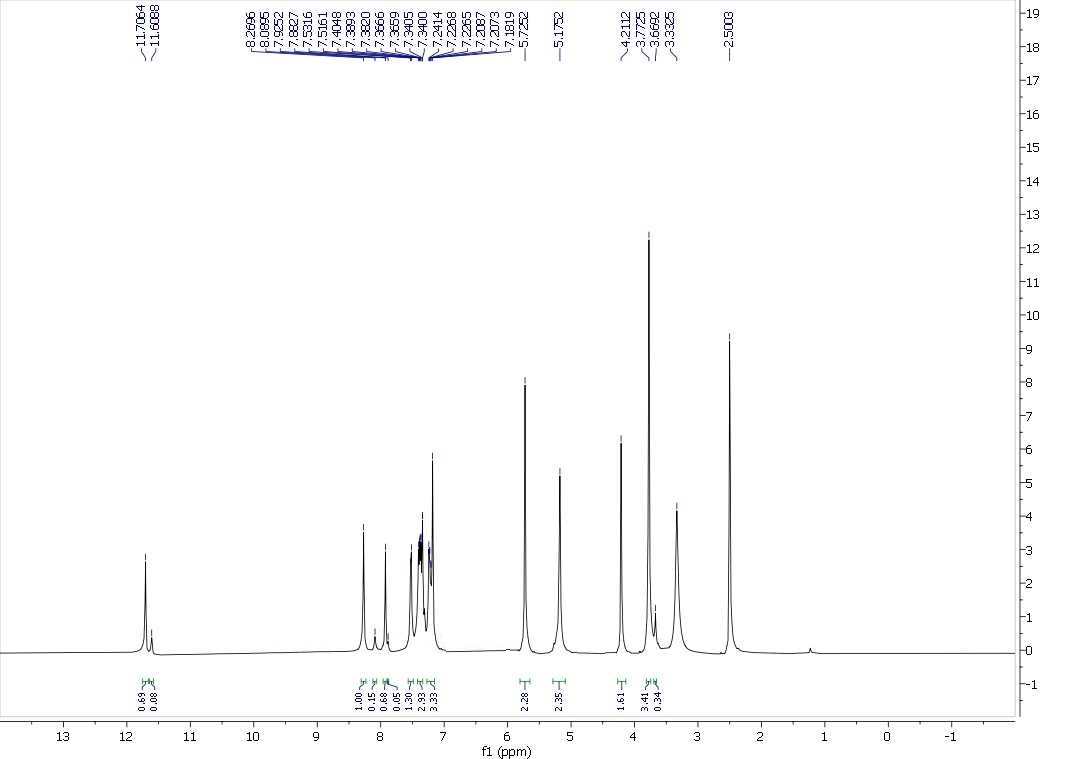


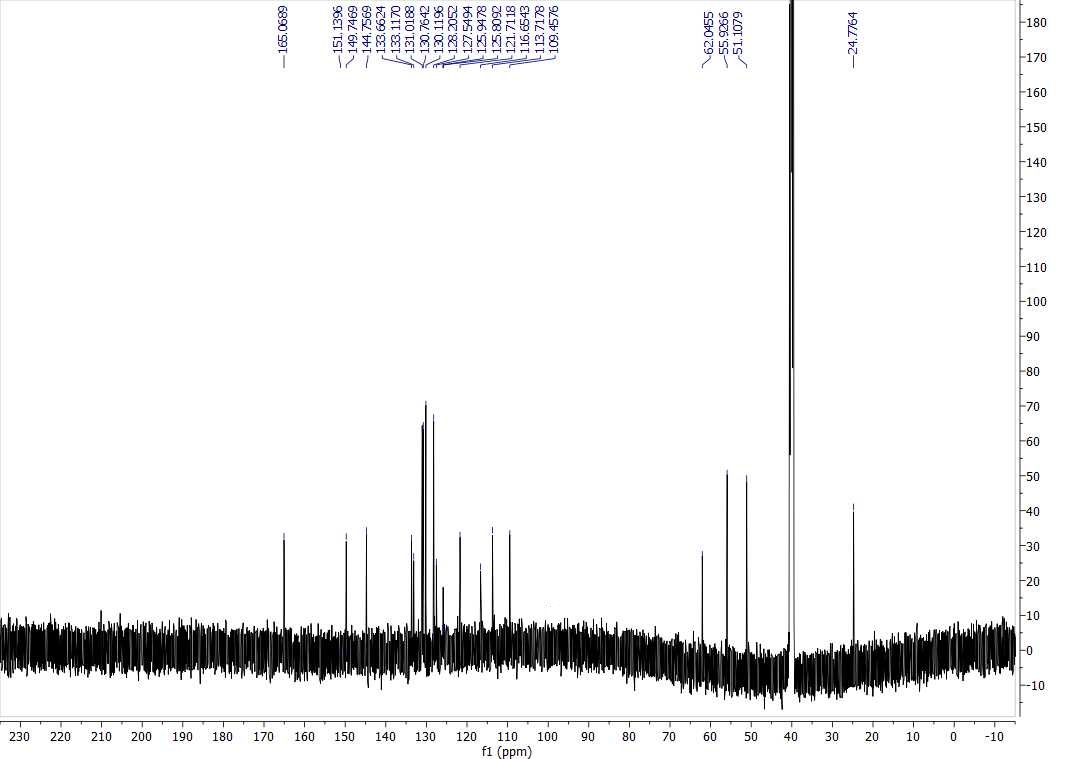


*N'-(4-((1-(4-Chlorobenzyl)-1H-1,2,3-triazol-4-yl)methoxy)-3-methoxybenzylidene)-2-cyanoacetohydrazide (****9e****)*

*
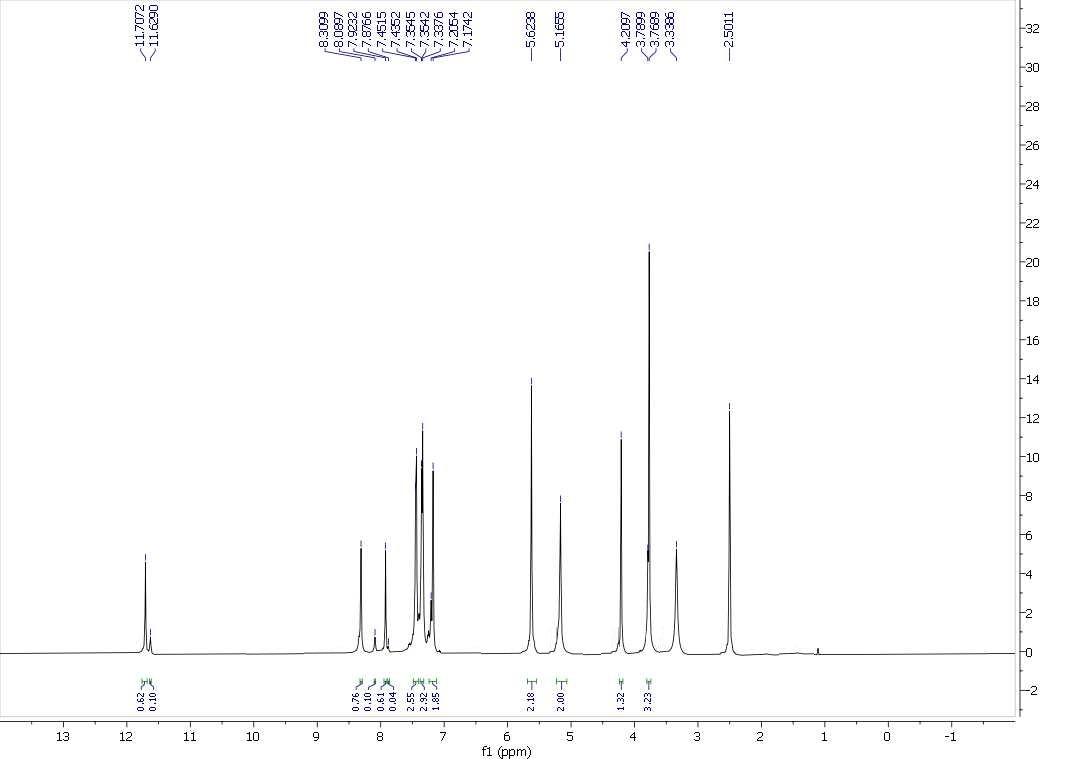
*


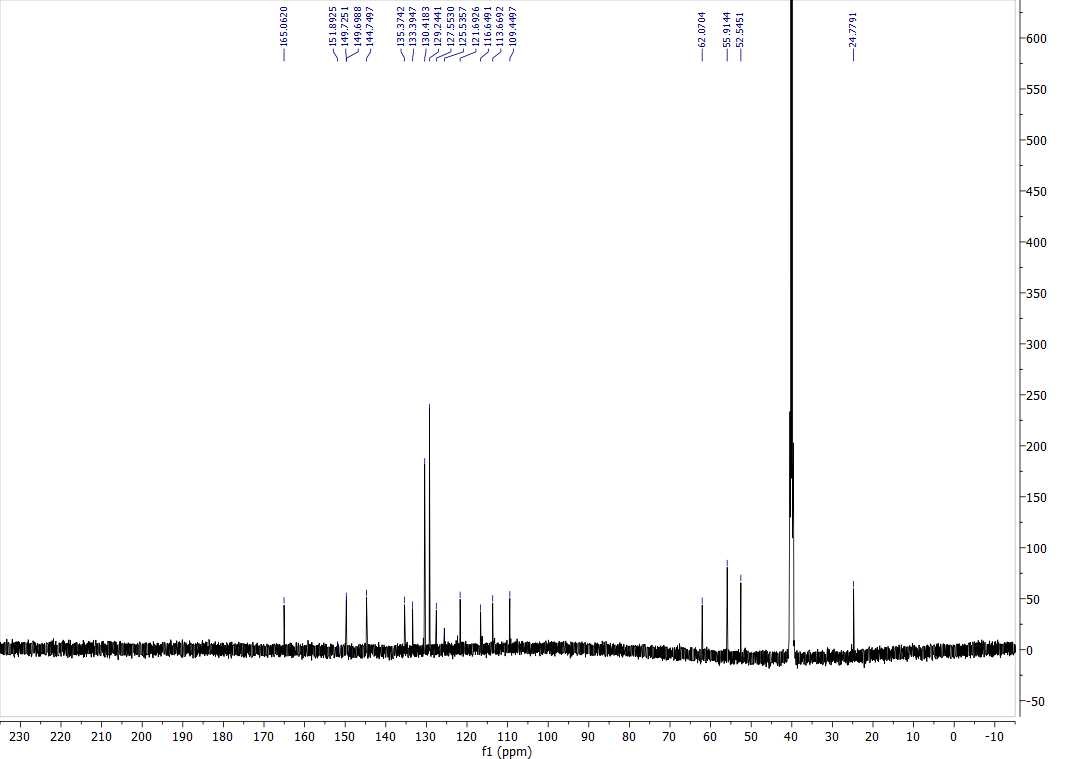


*2-Cyano-N'-(3-methoxy-4-((1-(2-methylbenzyl)-1H-1,2,3-triazol-4-yl)methoxy)benzylidene)acetohydrazide (****9f****)*

*
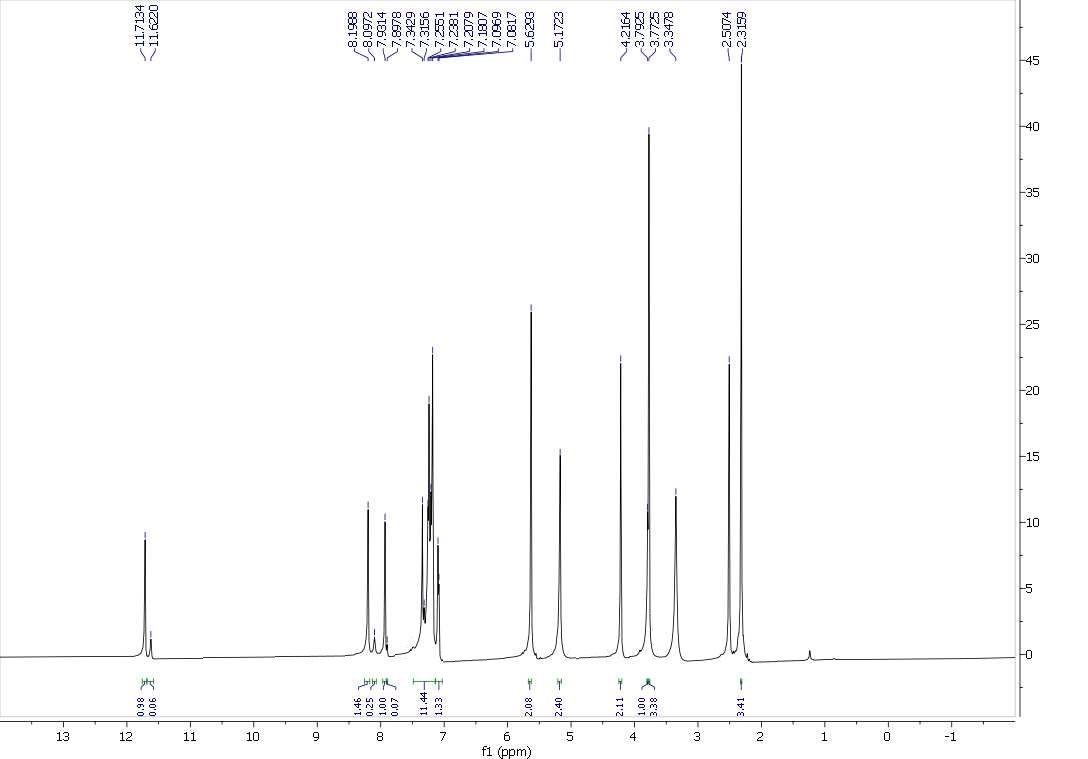
*


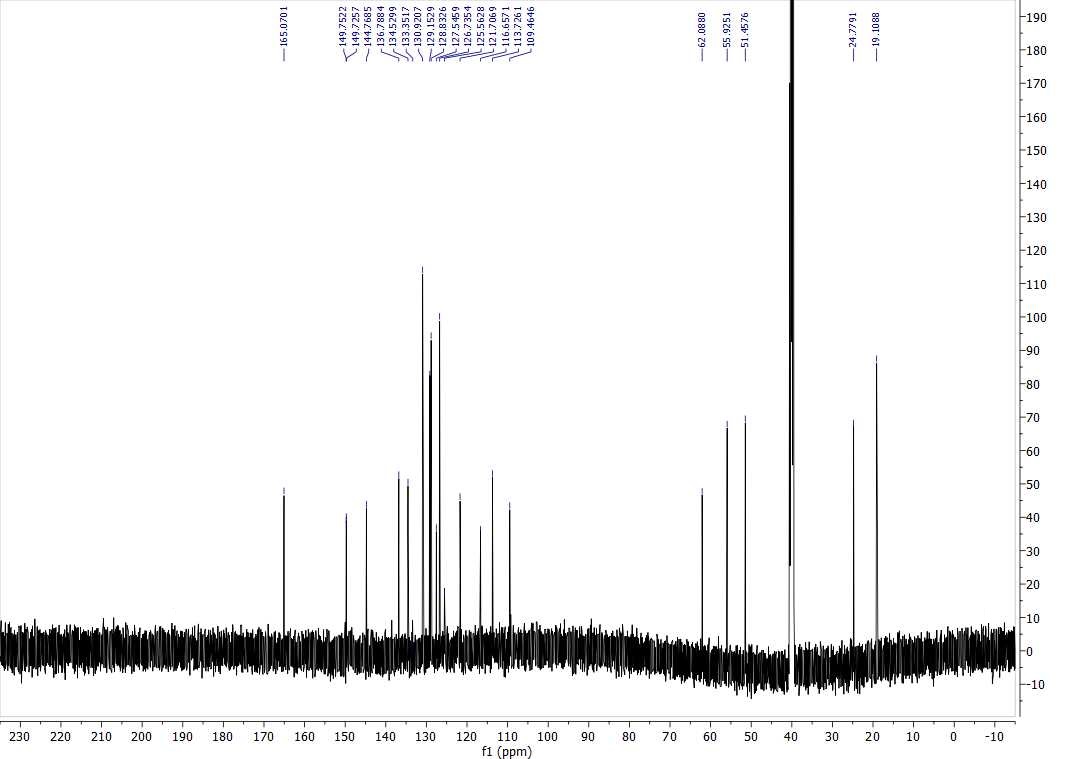


*2-Cyano-N'-(3-methoxy-4-((1-(4-methylbenzyl)-1H-1,2,3-triazol-4-yl)methoxy)benzylidene)acetohydrazide (****9g****)*

*
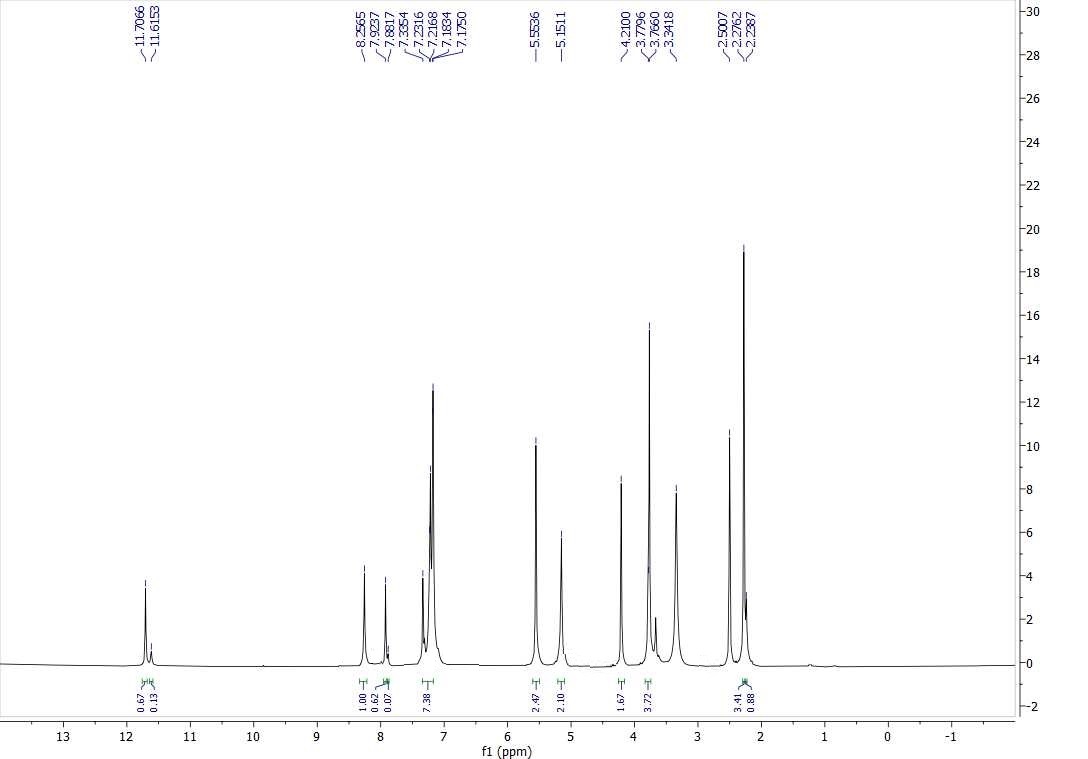
*


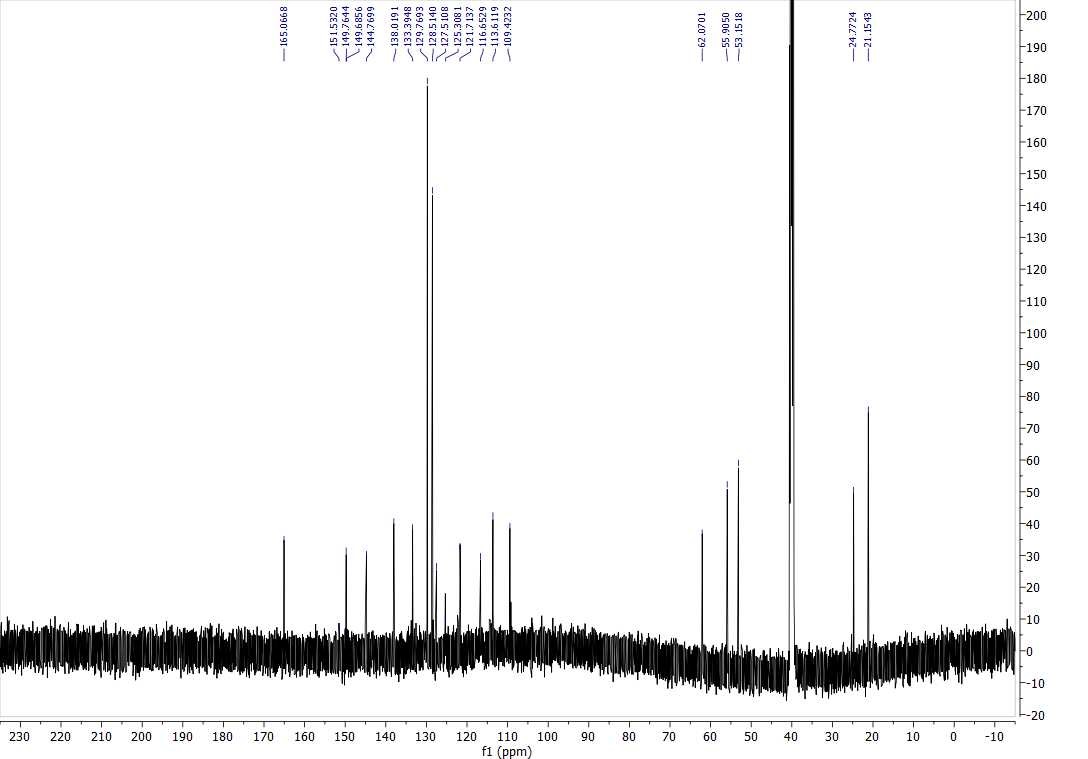


*N'-(4-((1-Benzyl-1H-1,2,3-triazol-4-yl)methoxy)benzylidene)-2-cyanoacetohydrazide (****9h****)*

*
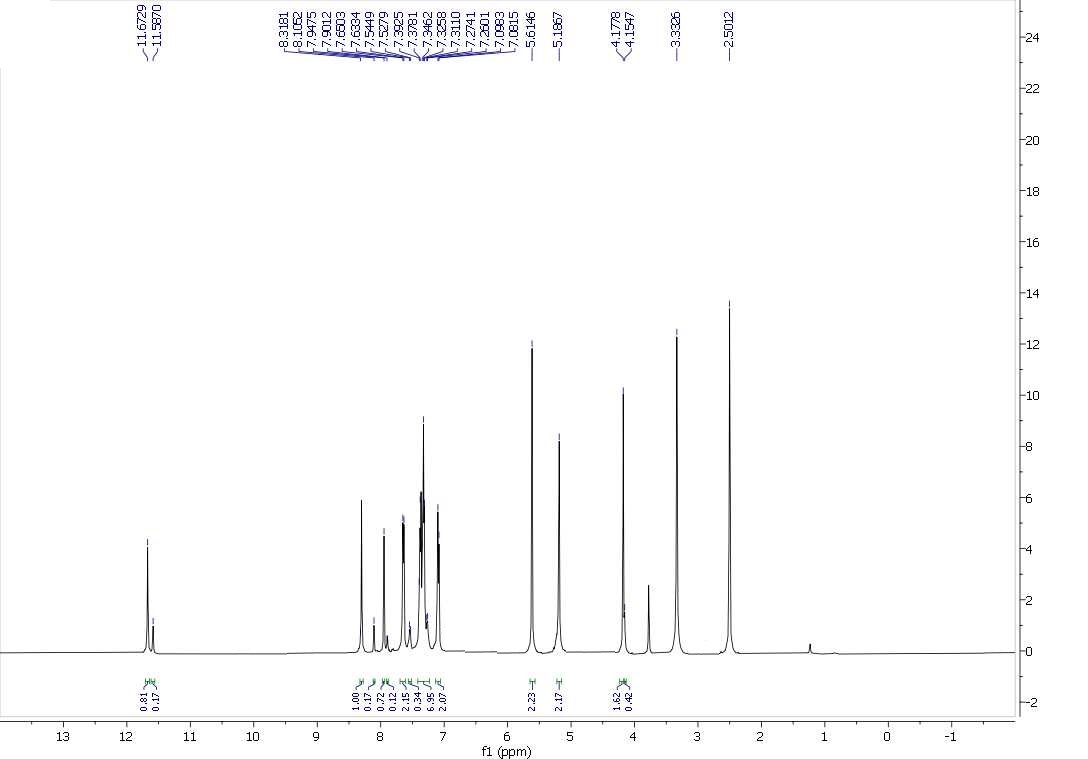
*


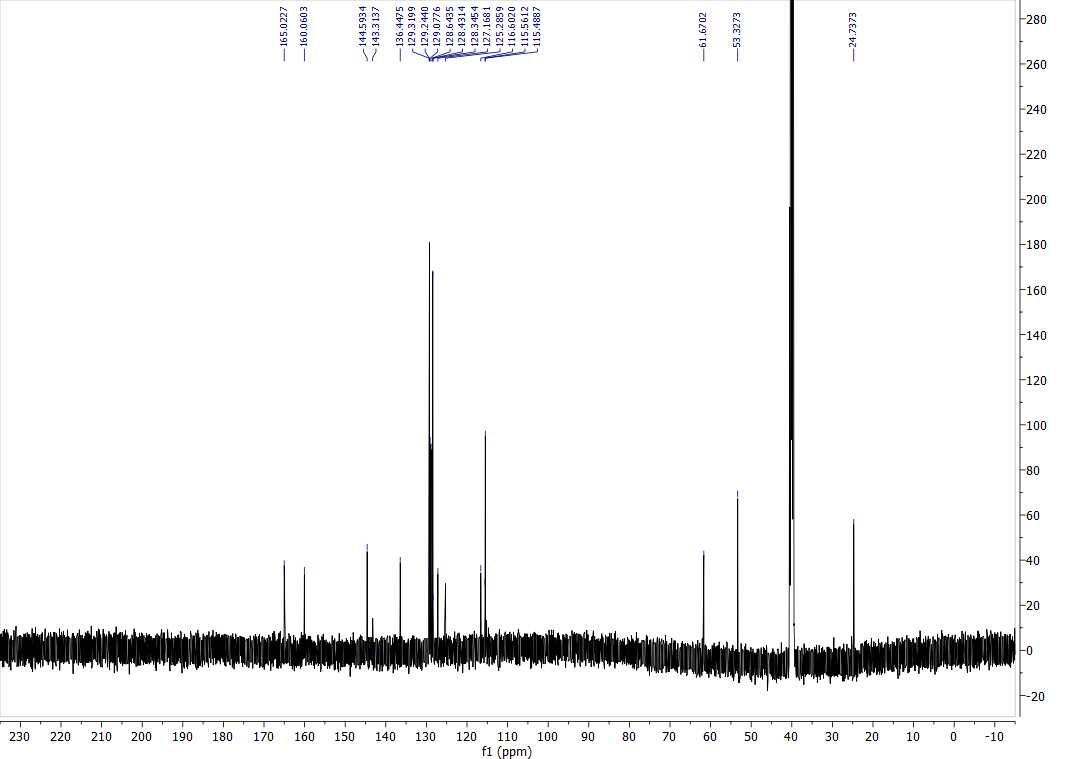


*2-Cyano-N'-(4-((1-(2-fluorobenzyl)-1H-1,2,3-triazol-4-yl)methoxy)benzylidene)acetohydrazide (****9i****)*

*
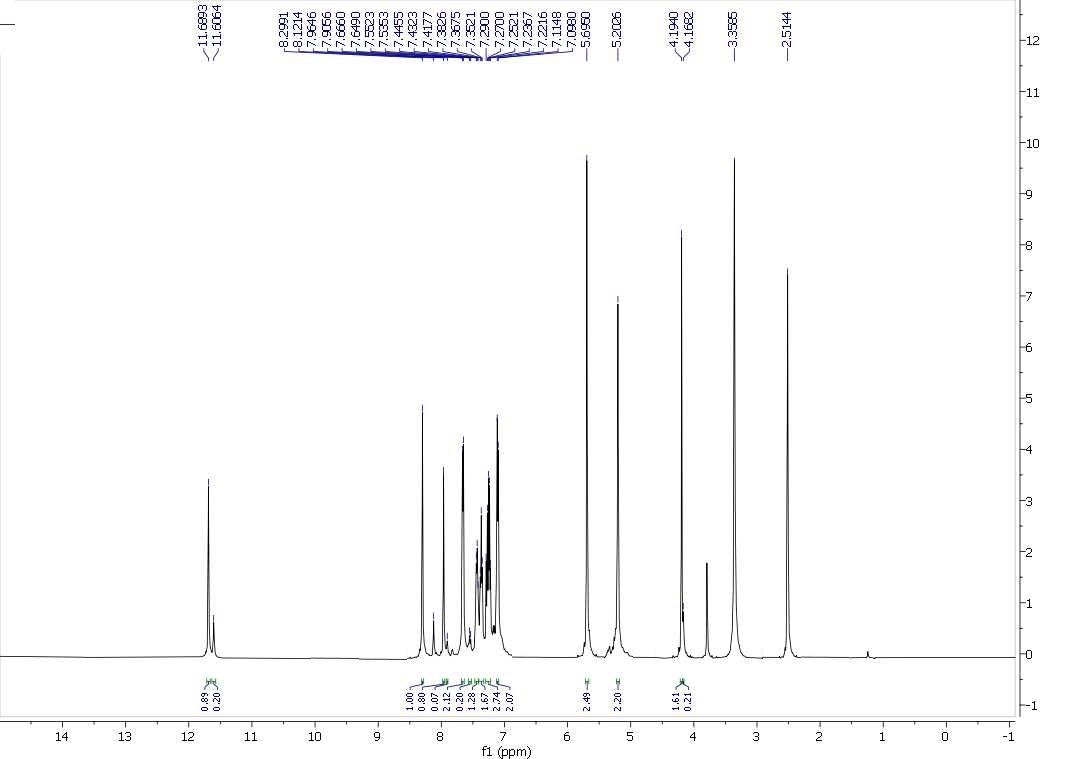
*


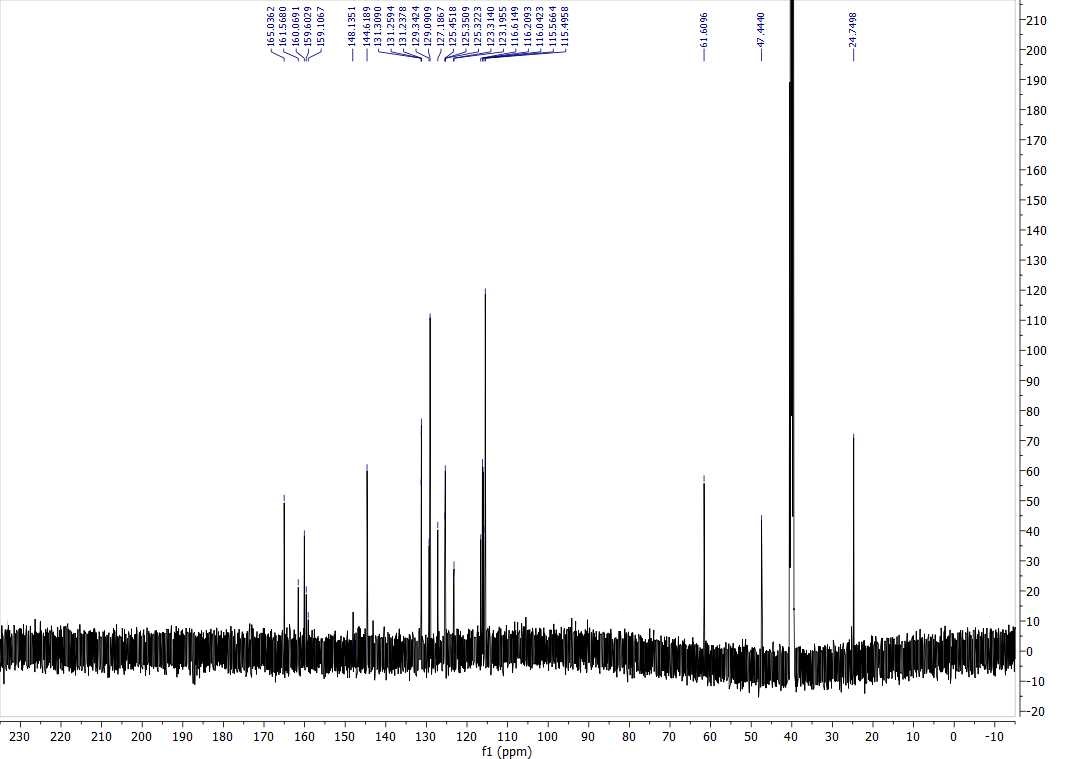


*2-Cyano-N'-(4-((1-(4-fluorobenzyl)-1H-1,2,3-triazol-4-yl)methoxy)benzylidene)acetohydrazide (****9j****)*

*
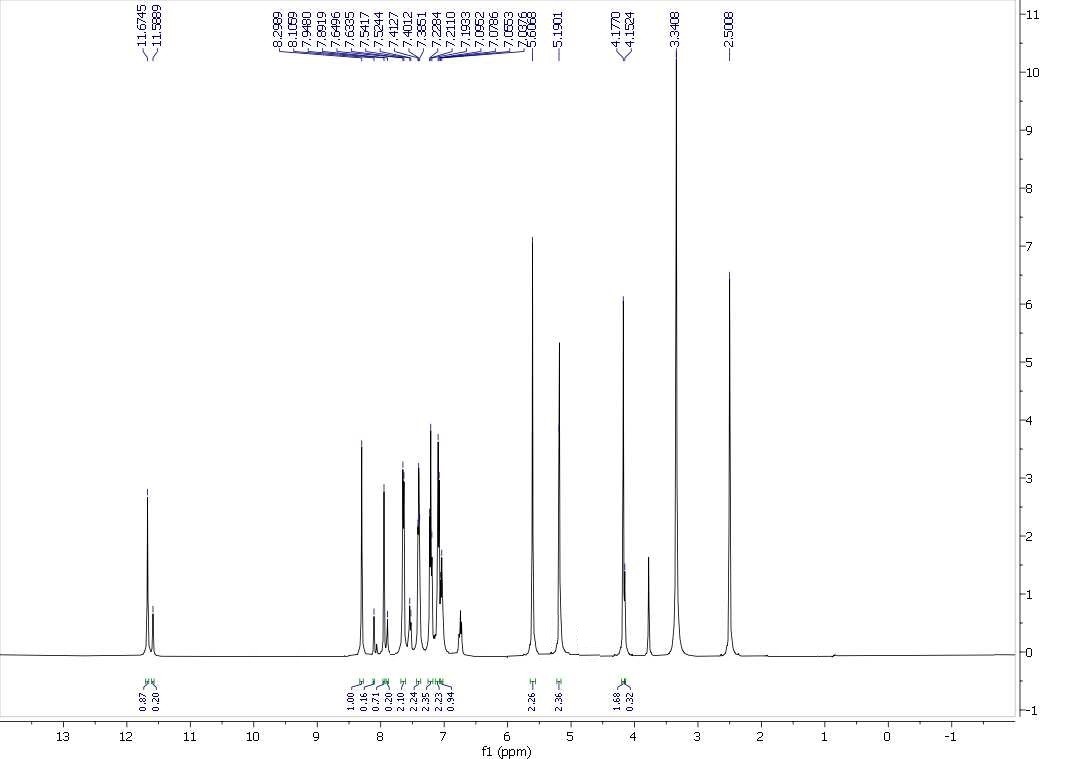
*


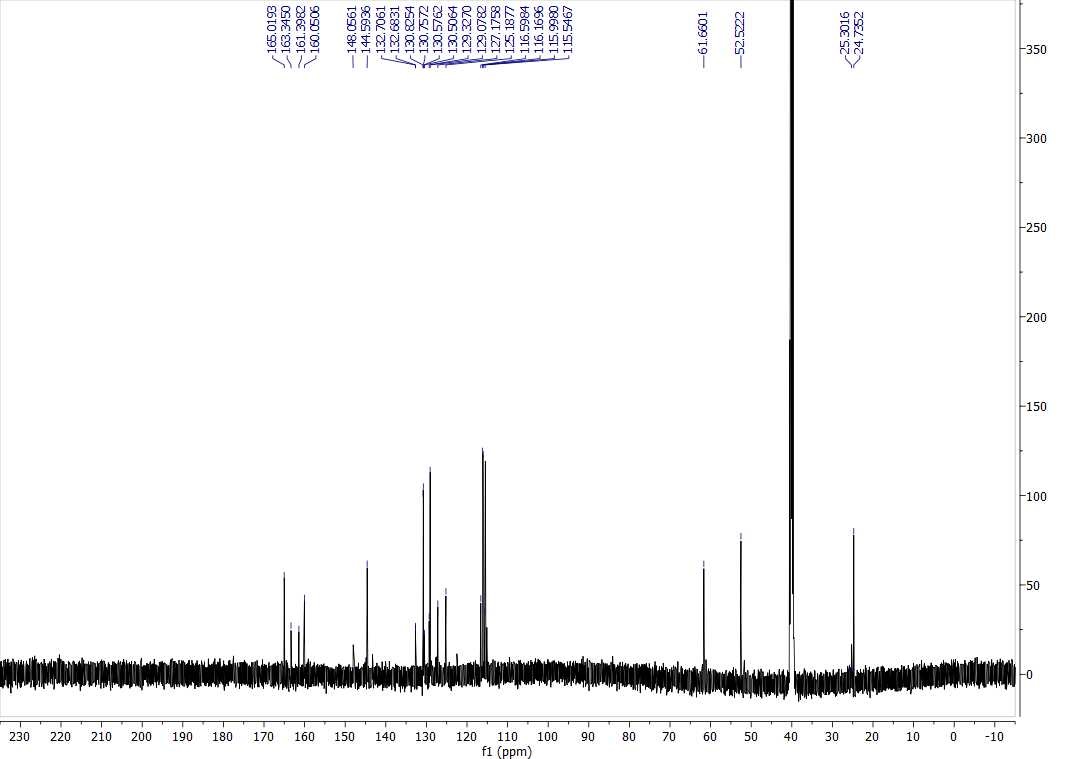


*N'-(4-((1-(2-Chlorobenzyl)-1H-1,2,3-triazol-4-yl)methoxy)benzylidene)-2-cyanoacetohydrazide (****9k****)*

*
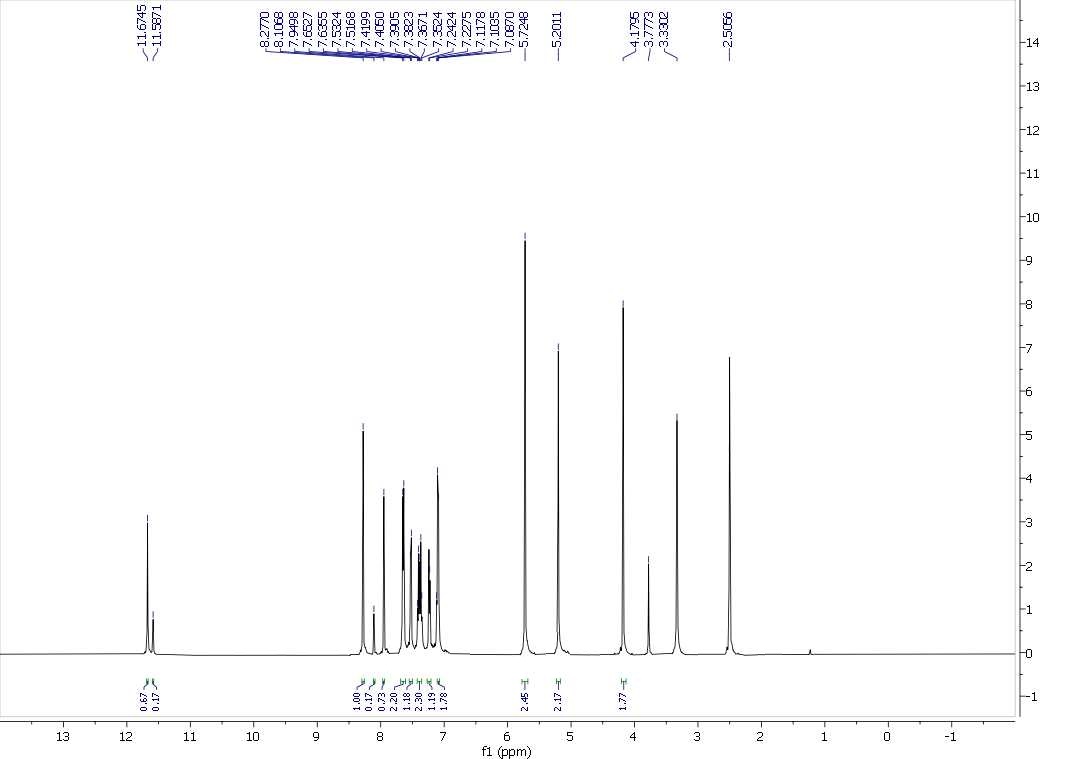
*


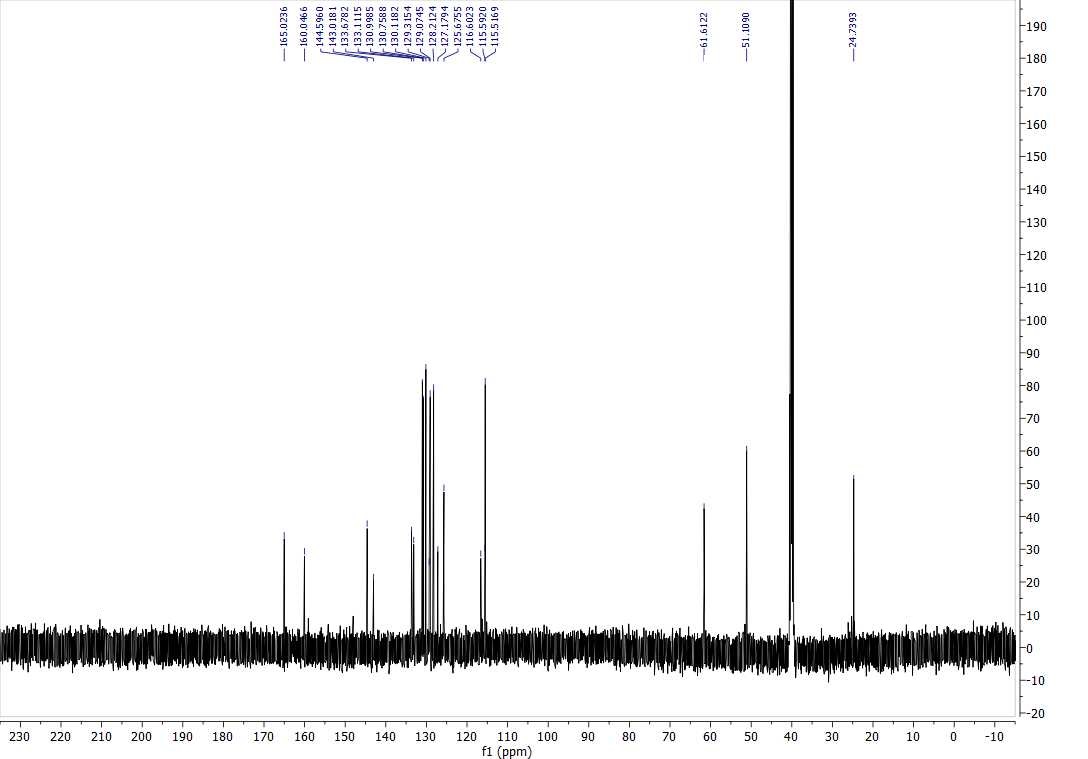


*N'-(4-((1-(4-Chlorobenzyl)-1H-1,2,3-triazol-4-yl)methoxy)benzylidene)-2-cyanoacetohydrazide (****9l****)*

*
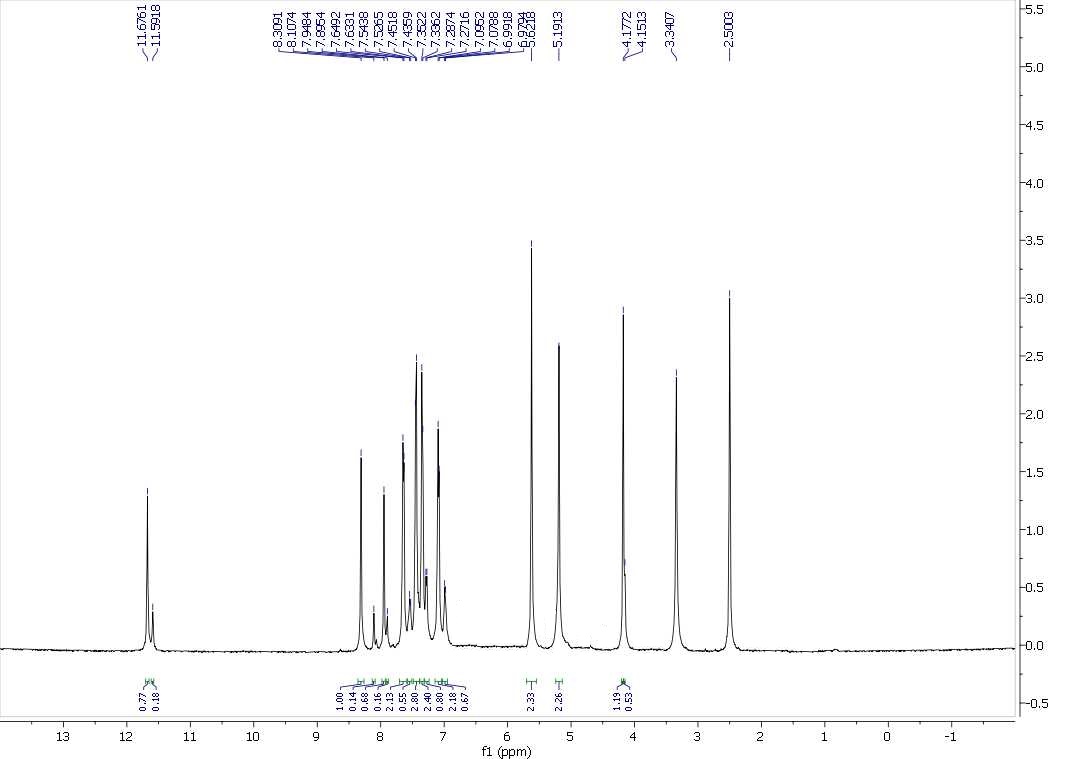
*


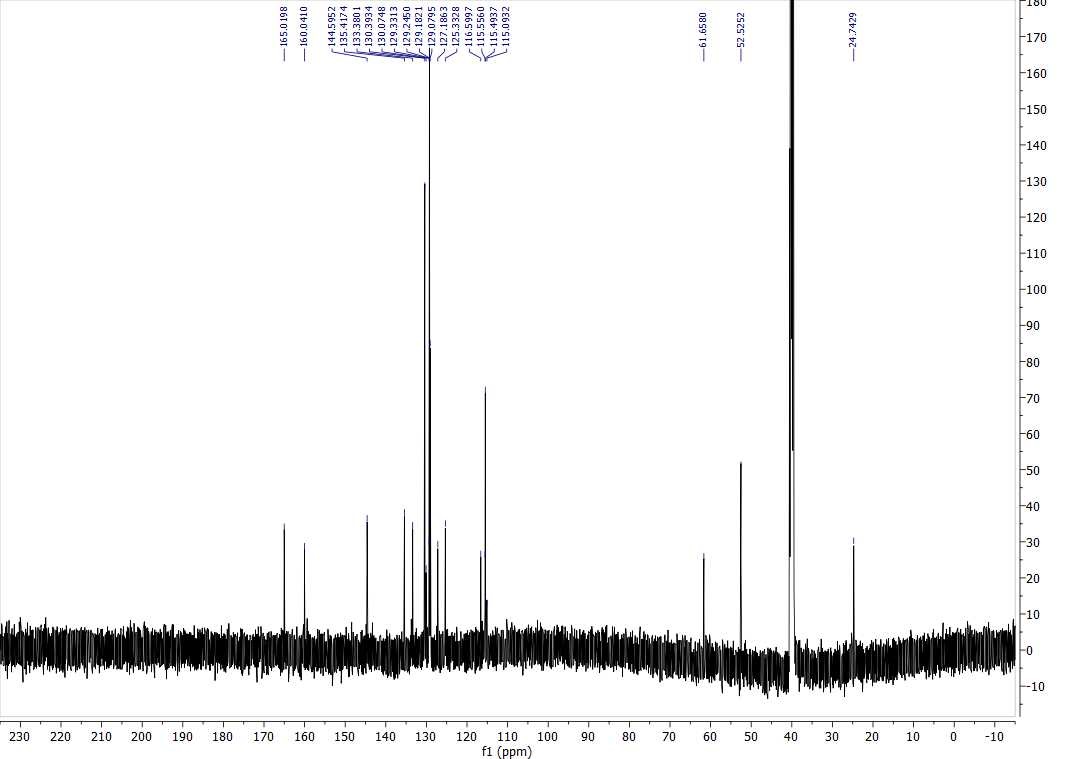


*2-Cyano-N'-(4-((1-(2-methylbenzyl)-1H-1,2,3-triazol-4-yl)methoxy)benzylidene)acetohydrazide (****9m****)*

*
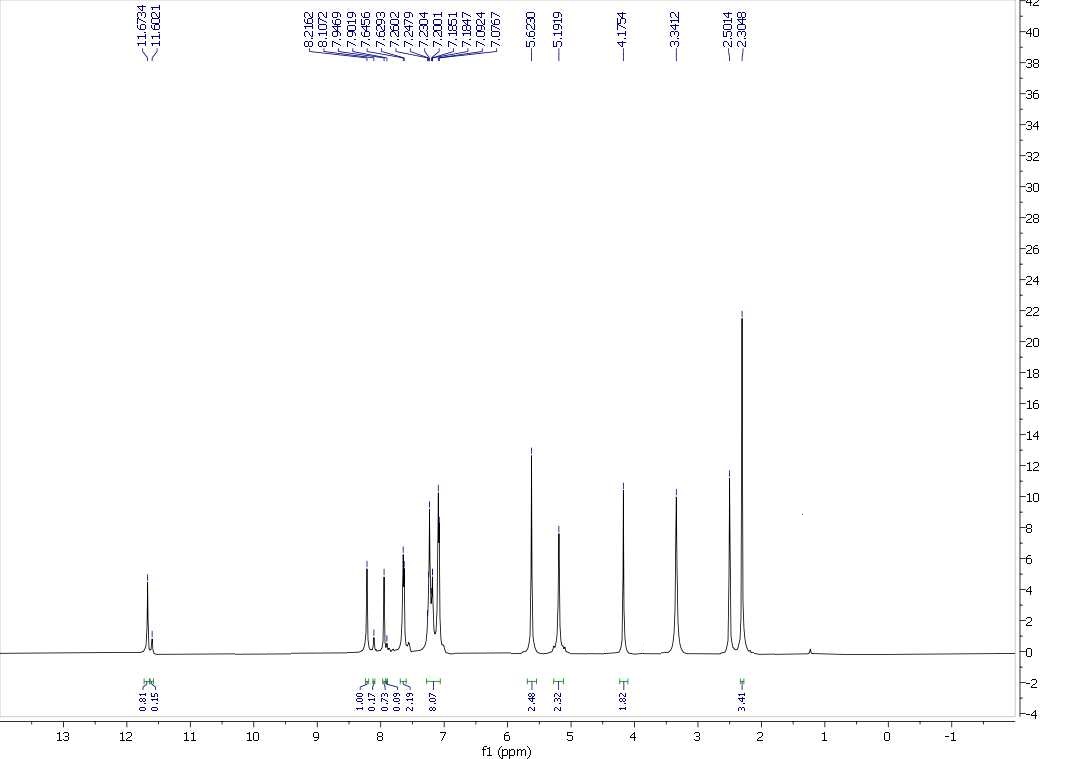
*


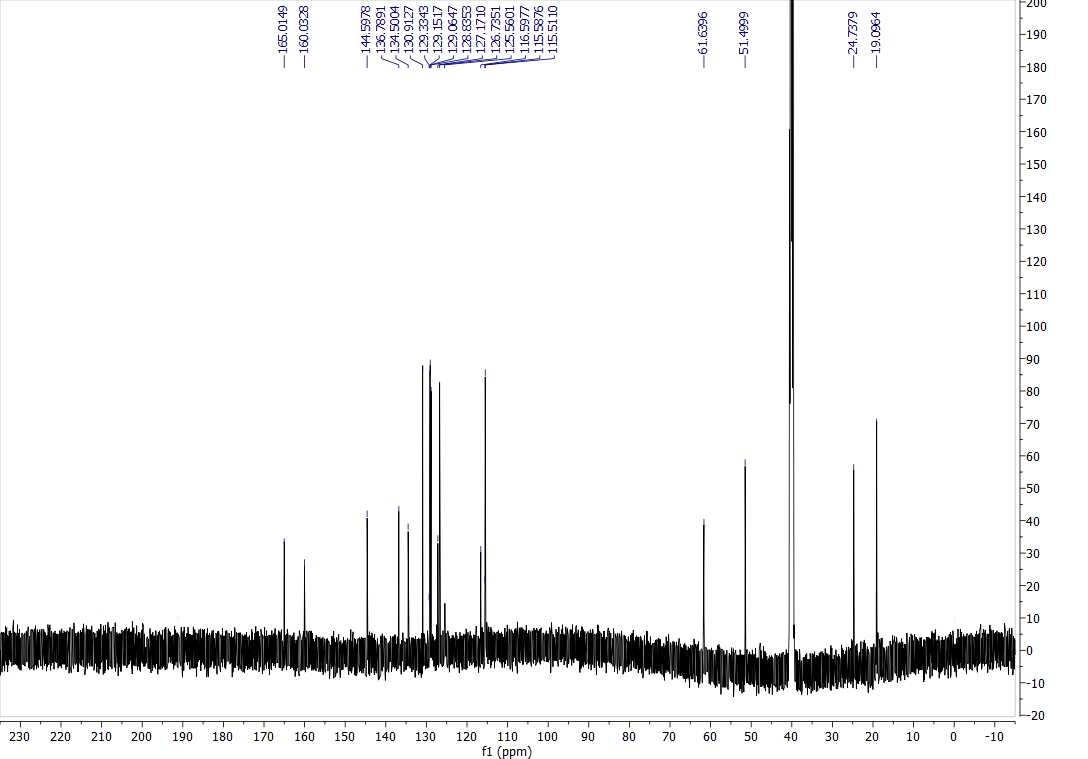


*2-Cyano-N'-(4-((1-(4-methylbenzyl)-1H-1,2,3-triazol-4-yl)methoxy)benzylidene)acetohydrazide (****9n****)*


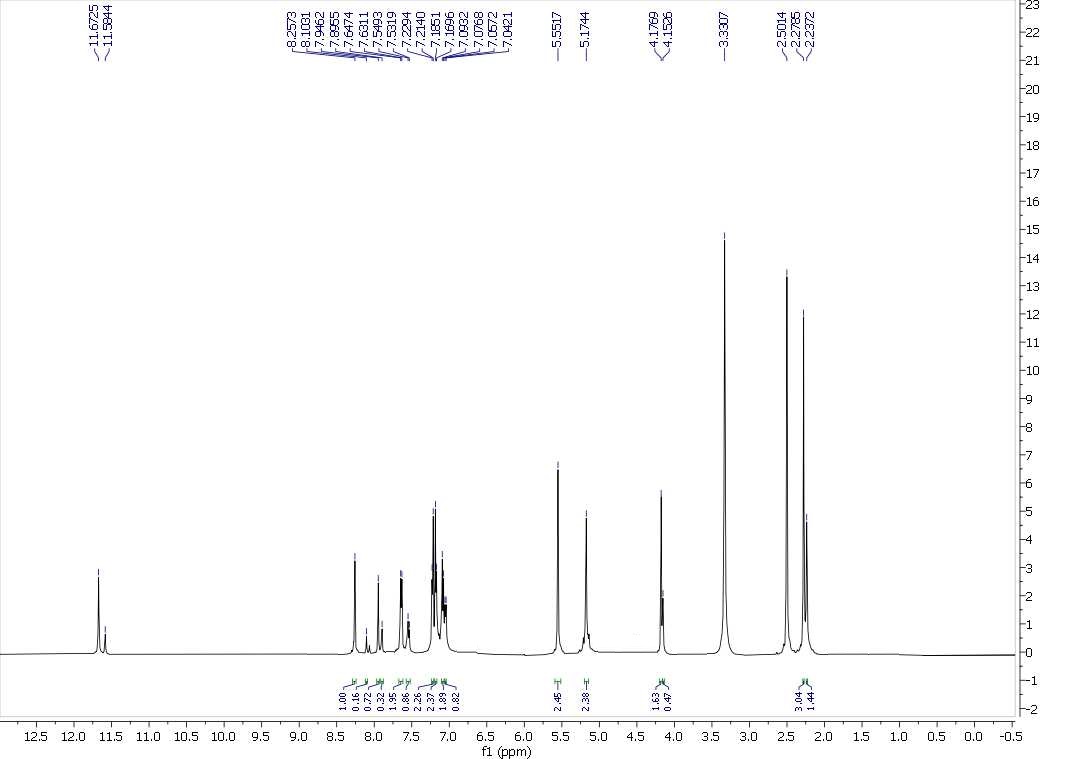


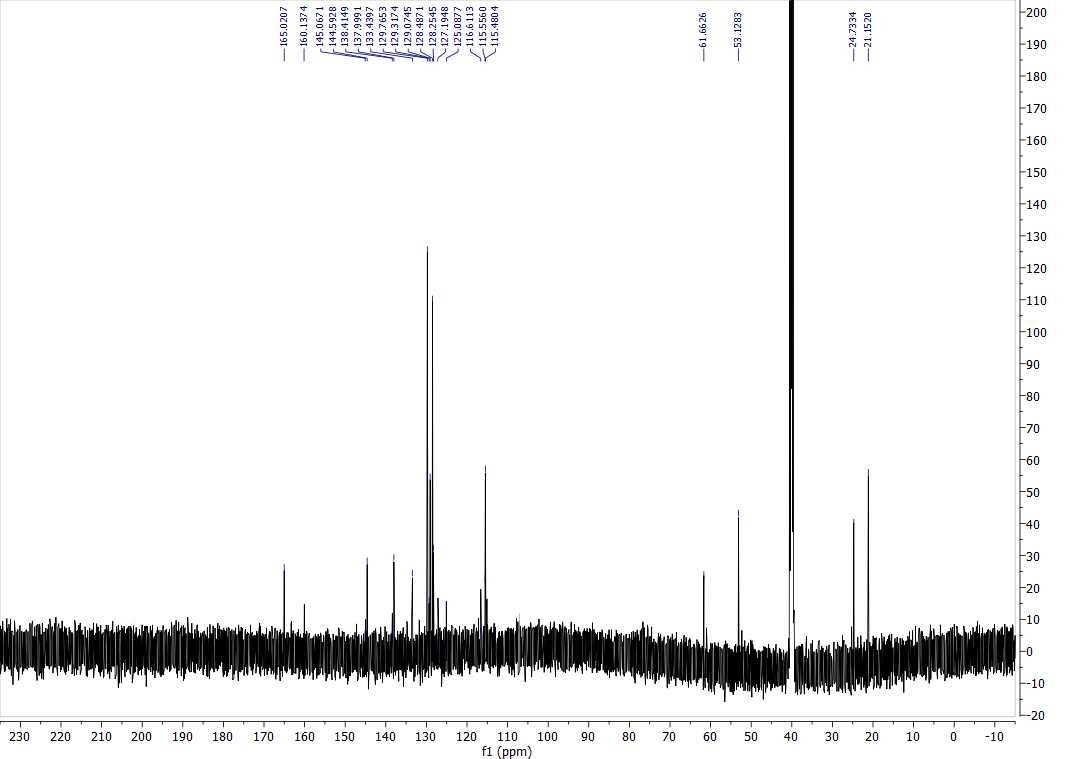


**HPLC chromatograms**

*Compound (****9a****)*

*
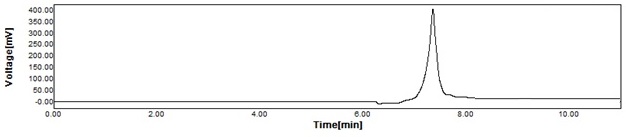
*

*Compound (****9b****)*

*
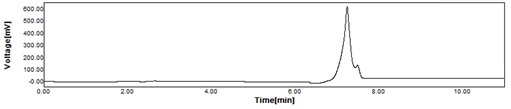
*

*Compound (****9c****)*

*
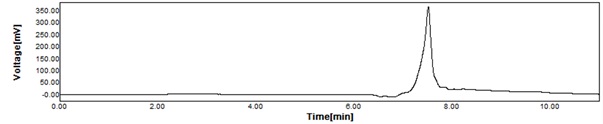
*

*Compound (****9d****)*

*
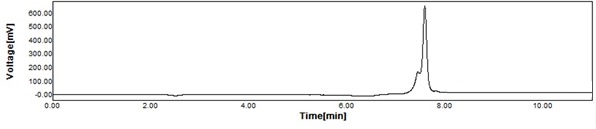
*

*Compound (****9e****)*

*
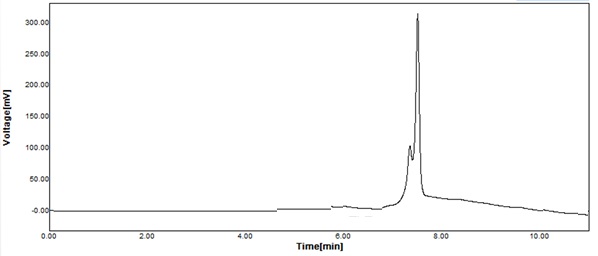
*

*Compound (****9f****)*

*
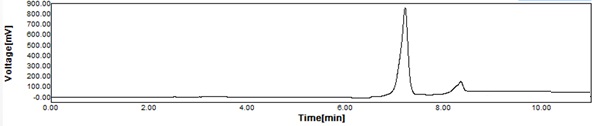
*

*Compound (****9g****)*

*
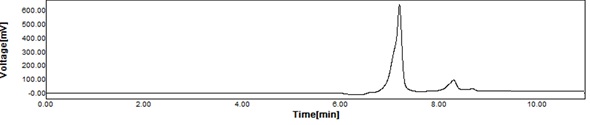
*

*Compound (****9h****)*

*
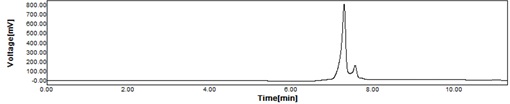
*

*Compound (****9i****)*

*
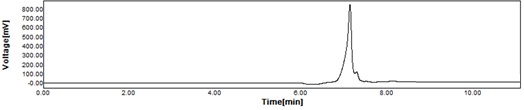
*

*Compound (****9j****)*

*
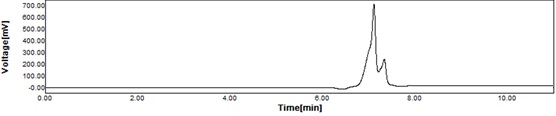
*

*Compound (****9k****)*

*
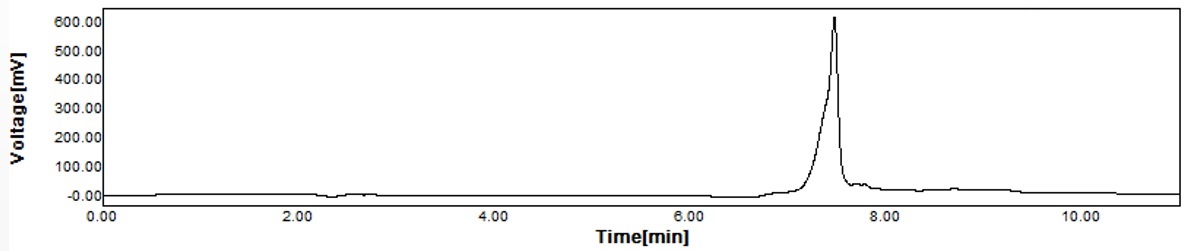
*

*Compound (****9l****)*

*
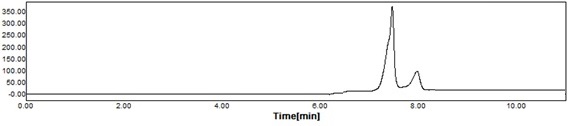
*

*Compound (****9m****)*

*
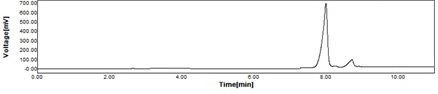
*

*Compound (****9n****)*

***
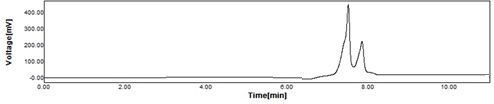
***

**CHN Analysis**
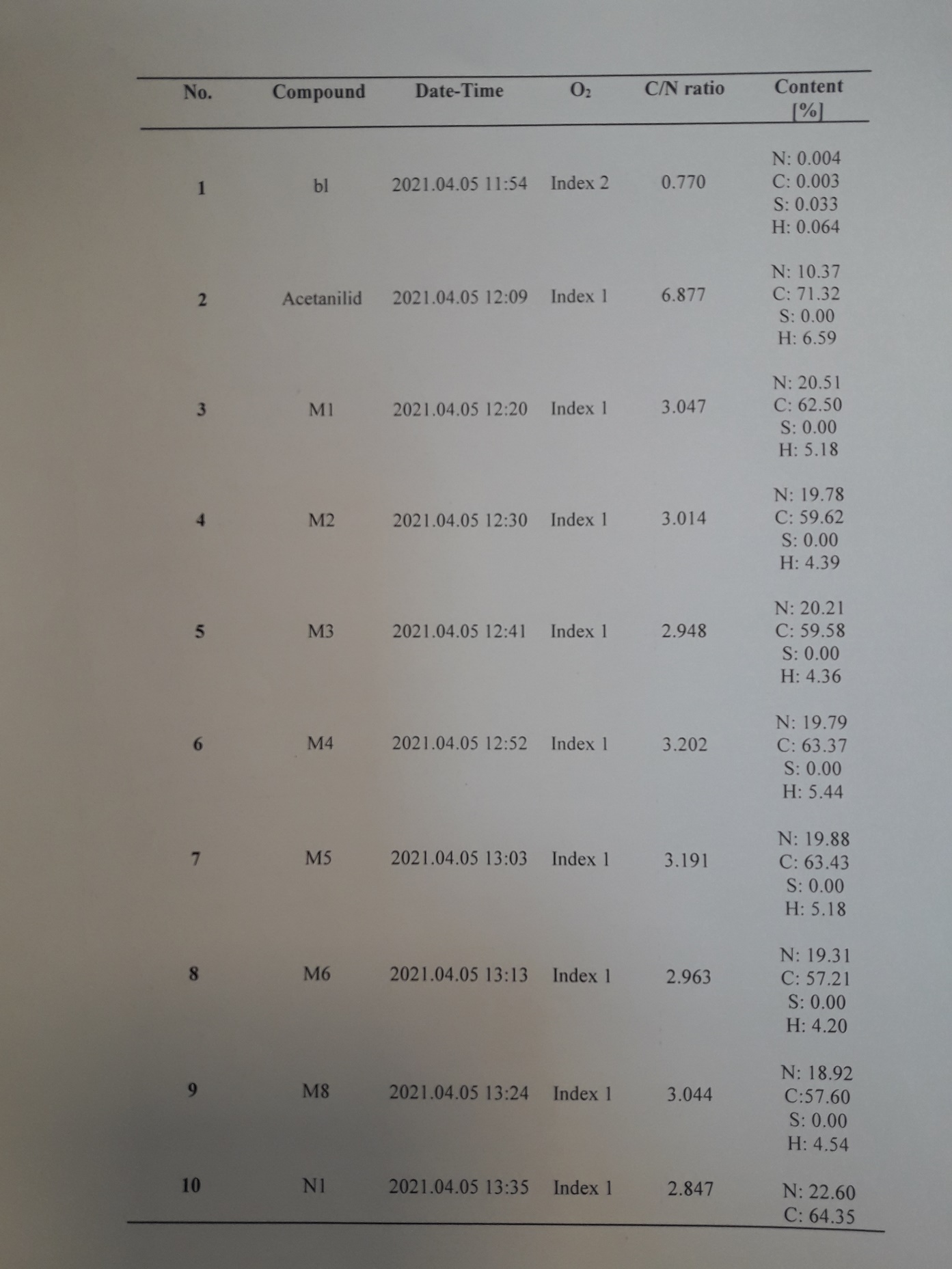


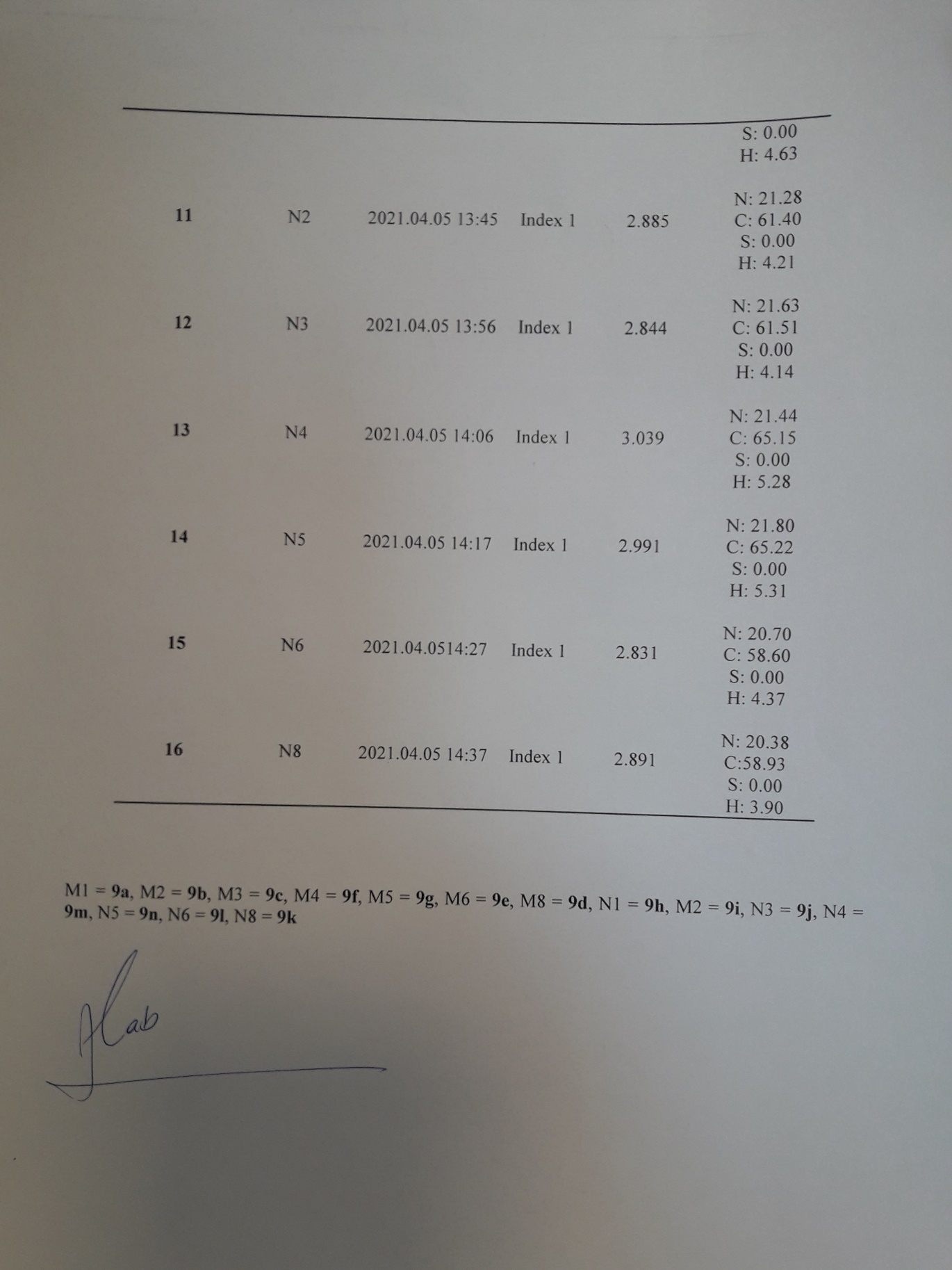

Supplement: Supplementary file 1 — Supplementary Information. [file 41598_2022_11771_MOESM1_ESM.docx]
